# Deletion of a core APC/C component reveals APC/C function in regulating neuronal USP1 levels and morphology

**DOI:** 10.3389/fnmol.2024.1352782

**Published:** 2024-06-12

**Authors:** Jennifer L. Day, Marilyn Tirard, Nils Brose

**Affiliations:** Department of Molecular Neurobiology, Max Planck Institute for Multidisciplinary Sciences, Göttingen, Germany

**Keywords:** SUMO, ubiquitin, E3 ligase, protein degradation, neuron, APC/C, USP1, Anaphase Promoting Complex

## Abstract

**Introduction:**

The Anaphase Promoting Complex (APC/C), an E3 ubiquitin ligase, plays a key role in cell cycle control, but it is also thought to operate in postmitotic neurons. Most studies linking APC/C function to neuron biology employed perturbations of the APC/C activators, cell division cycle protein 20 (Cdc20) and Cdc20 homologue 1 (Cdh1). However, multiple lines of evidence indicate that Cdh1 and Cdc20 can function in APC/C-independent contexts, so that the effects of their perturbation cannot strictly be linked to APC/C function.

**Methods:**

We therefore deleted the gene encoding Anaphase Promoting Complex 4 (APC4), a core APC/C component, in neurons cultured from conditional knockout (cKO) mice.

**Results:**

Our data indicate that several previously published substrates are actually not APC/C substrates, whereas ubiquitin specific peptidase 1 (USP1) protein levels are altered in APC4 knockout (KO) neurons. We propose a model where the APC/C ubiquitylates USP1 early in development, but later ubiquitylates a substrate that directly or indirectly stabilizes USP1. We further discovered a novel role of the APC/C in regulating the number of neurites exiting somata, but we were unable to confirm prior data indicating that the APC/C regulates neurite length, neurite complexity, and synaptogenesis. Finally, we show that APC4 SUMOylation does not impact the ability of the APC/C to control the number of primary neurites or USP1 protein levels.

**Discussion:**

Our data indicate that perturbation studies aimed at dissecting APC/C biology must focus on core APC/C components rather than the APC/C activators, Cdh20 and Cdh1.

## 1 Introduction

A complex array of post-translational modifications regulates many simultaneously active signaling pathways within cells, thereby controlling cellular physiology (Chen et al., 2017). One class of such modifications involves a cascade of E1, E2, and E3 enzymes to covalently attach ubiquitin or small ubiquitin-like modifier (SUMO) to lysine residues of substrates. Ubiquitin itself contains seven different lysine residues that each can be ubiquitylated, allowing for the formation of diverse chain types that have a variety of functions. In some cases, ubiquitylation leads to substrate degradation by the proteasome, and in this way regulates complex signaling networks by triggering spatiotemporal selective protein degradation (Oh et al., 2018).

The APC/C is an E3 ubiquitin ligase that stands out as a regulator of complex signaling networks. Upon activation by Cdc20 or Cdh1, the APC/C regulates the cell cycle via oscillating ubiquitylation of defined sets of proteins. The APC/C is a large complex of at least 11 distinct proteins (Peters, 2006), most of which are also expressed in non-dividing neurons, indicating functions of the APC/C beyond cell cycle control (Gieffers et al., 1999). Accordingly, the APC/C has been implicated in the regulation of a variety of substrates and processes in neurons, from glycolysis to synaptogenesis (Eguren et al., 2011). However, the corresponding studies involved the perturbation of the APC/C activators and not core APC/C components. Indeed, deletion of the core APC/C component, APC2, from excitatory mouse forebrain neurons does not alter levels of Ski-novel protein (SnoN) and GluA1 (Kuczera et al., 2011). While this unexpected finding might be due to the fact that the APC/C ubiquitylates substrates only transiently, in certain cells, and under specific conditions, an alternative explanation is that Cdh1 and Cdc20 have APC/C-independent “moonlighting” functions (Wan et al., 2011, 2017; Liu et al., 2016; Han et al., 2019), so that certain phenotypes caused by Cdh1 or Cdc20 depletion are unrelated to APC/C function.

In accordance with this notion, Cdh1 and Cdc20 operate independently of the APC/C to regulate protein stability and protein–protein interactions. For instance, Cdh1 regulates SMURF1 protein levels and dimerization through a mechanism that requires a D-box but is independent of the APC/C (Wan et al., 2011; Kannan et al., 2012). Similarly, APC/C-independent binding of Cdh1 to a D-box motif on c-Src inhibits its kinase activity (Han et al., 2019). Cdh1 employs an APC/C-independent mechanism to suppress dimerization and kinase activity of BRAF (Wan et al., 2017), and it suppresses the auto-ubiquitylation of WWP2, increasing its activity (Liu et al., 2016). Finally, Parkin, an E3 ubiquitin ligase involved in the development of Parkinson’s disease, binds to both Cdh1 and Cdc20 independently, but not to core APC/C components, and this association regulates APC/C substrates and the cell cycle. A double knockdown of Parkin and core APC/C components are required to phenocopy a Cdh1 knockdown (Lee et al., 2015), but it is unclear if Parkin and Cdh1 also interact in neurons.

Our study was designed to directly explore the requirement for APC/C activity in nerve cell development and function. We inactivated the APC/C by genetically eliminating the core APC/C component, APC4, in cultured neurons and determined the effects on previously-proposed APC/C substrates and related phenotypes. In contrast to prior studies employing activator depletion (Eguren et al., 2011), we show that neither SnoN, NEUROD2, and FEZ1 levels, nor, synaptogenesis and neurite length and branching are affected by APC/C-inactivation. Instead, we provide evidence for a temporally-regulated pathway where the neuronal APC/C controls USP1 levels and the number of neurites exiting neuron somata. We demonstrate that these phenotypic changes are rescued by APC4 re-expression, and that APC4 SUMOylation does not alter APC/C-dependent regulation of neuron morphology and USP1 protein levels. Our data indicate that care must be taken when extrapolating APC/C function from experimental data obtained by Cdh1 or Cdc20 perturbation. Finally, we show that the APC/C has a detectable but subordinate role in early nerve cell development, and that USP1 may be an important neuronal APC/C substrate.

## 2 Materials and methods

### 2.1 Animals

All mice were in the C57/N background. Mutant lines are listed in Supplementary Table S1. For harvesting of tissue, isoflurane-anesthetized adult mice were killed by cervical dislocation, and P0 pups and E16 embryos were killed by decapitation. Mice were housed in individually ventilated cages at ambient temperature under a 12 h light/dark cycle, with free access to food and water. The sex of mice used for cell cultures was not determined. For genotyping, DNA was isolated from tail biopsies using a genomic DNA isolation kit (Nexttec, #10.924). Genotyping primers and PCR product sizes are listed in Supplementary Table S2. The genotyping PCR reaction (96°C for 3 min; 42 cycles of 94°C for 30 s, 62°C for 1 min and 72°C for 1 min; 72°C for 7 min) included 0.05 U/μL MyTaq HS DNA Polymerase (Biotool, #BIO-21113), MyTaq reaction buffer, 1 mM dNTPs, 0.2 nM primers, and 1 μL tail DNA (~15–80 ng DNA).

### 2.2 Plasmids

PCR was used to generate sequences encoding N-terminally HA and Myc tagged APC4. Resulting constructs were cloned into pcDNA3.1 or lentiviral vectors (kind gifts of C. Rosenmund) that drive APC4 expression with the neuron-specific Synapsin1 promoter. Cre NLS RFP and NLS RFP vectors are in the pf(syn)w-rbn lentivirus backbone. The lentiviral vector pf(syn)w-iCreRFP-P2A expresses iCre-RFP fused to a self-cleaving P2A sequence. This vector was used to generate the APC4 rescue vectors by cloning APC4 after the P2A sequence. APC4 lysines 772 and 797 were mutated to arginine using site directed mutagenesis. The EGFP pCS2+ control plasmid was cloned by replacing Cdc20 with EGFP. Supplementary Table S3 lists the remaining constructs.

### 2.3 Cell culture

HEK293FT cells (Invitrogen) were maintained at 37°C and 5% CO_2_ in DMEM containing 10% FBS (Gibco) and 50 units/mL penicillin/streptomycin (Gibco). For overexpression experiments, cells were transfected using Lipofectamine 2000 (ThermoFisher). Primary neuron cultures were generated as described previously (Daniel et al., 2017). P0 hippocampi and E16 cortices were digested for 30–60 min at 37°C using DMEM containing papain (25 units/mL; Worthington Biochemical), 0.2 mg/mL L-cysteine (Sigma), 1 mM CaCl_2_, and 0.5 mM EDTA (in DMEM). Dissociated neurons were plated on poly-L-lysine-coated glass coverslips (Thermo, 12 mm coverslips #1.0) or dishes (Sigma). Neurons were cultured at 37°C with 5% CO_2_ in Neurobasal-A medium supplemented with 2% B27, penicillin/streptomycin, and 1% GlutaMAX-1 (Gibco). For imaging, cells were seeded on glass coverslips in a 24-well plate at 25,000–50,000 cells/well. For biochemical analysis, ~1.2 million cells were seeded per well of a 6-well plate, and the media was changed the next day to promote cell survival.

### 2.4 Lentivirus transduction

Lentivirus was prepared using standard methods (López-Murcia et al., 2019). HEK293 cells were plated on 15 cm dishes coated with poly-L-lysine (Sigma) and grown in standard HEK293 cell media containing 0.4 μg/μL Geneticin (Gibco). Immediately before transfection, when cells were 90% confluent, the media was changed to Opti-MEM with 10% FBS (Gibco). All vectors (expression, envelope, and packaging) were co-transfected in Opti-MEM with Lipofectamine 2000 (Invitrogen). After 6 h, the media was changed to pre-warmed media (DMEM, Gibco; 1% Penn/Strep, Gibco; 2% goat Serum, Gibco; 10 mM sodium butyrate, Merck). The virus was harvested after 44–48 h and concentrated with Amicon centrifugal filters (100 kDa; Millipore). Flash-frozen aliquots were stored at-80°C. The percentage of neurons co-expressing MAP2, RFP, and DAPI was used to determine virus titer. Infection rates were typically >90%.

### 2.5 Primary antibodies

Supplementary Table S4 lists the primary antibodies used.

### 2.6 Secondary antibodies

Supplementary Table S5 lists the secondary antibodies used.

### 2.7 Immunolabeling

Coverslips seeded with neurons were washed 3 times with PBS, fixed for 10 min (4% PFA; Serva), washed four times, and blocked for 30 min in imaging solution (0.1% fish skin gelatin, Sigma; 1% goat serum, Gibco; 0.3% Triton X-100, Roche; PBS; Daniel et al., 2017). Coverslips were incubated with primary antibodies in imaging solution for 16–21 h at 4°C. After washing, coverslips were incubated with secondary antibodies for 1 h in imaging solution. Coverslips were then incubated with DAPI (Thermo; 1:10,000) for 10 min, washed with PBS, and mounted onto slides with Aqua-Poly/Mount (Polyscience Inc.).

### 2.8 Western blotting

Neurons were lysed in lysis buffer (150 mM NaCl; 10 mM Tris pH 7.4; 1% Triton X-100) containing protease inhibitors (1 μg/mL aprotinin, Roche; 0.5 μg/mL leupeptine, Roche; 17.4 μg/mL PMSF, Roche), and fresh N-ethylmaleimide (NEM; 20 mM, Sigma) when required. Protein concentration was determined by the BCA method (Pierce). Typically, 20–25 μg of protein were loaded per lane of SDS/PAGE gel (40 μg were required for detecting Cyclin B1). After SDS-PAGE (Laemmli, 1970), samples were transferred to Nitrocellulose membranes (0.2 mm NC; Amersham Protran, #1060001; Towbin et al., 1979). To assess transfer, membranes were stained with MemCode or Ponceau S. WB was conducted using standard procedures (Daniel et al., 2017). Blocking and antibody incubation were done in PBS with 5% milk powder and 1% Tween. Signals were developed by enhanced chemiluminescence (GE Healthcare) and detected with a Chemostar Imager (INTAS Science Imaging) or, in some cases, by photographic film. For some experiments (Supplementary Figures S3A–D), WB was performed with fluorescent secondary antibodies and signals were detected by an Odyssey Infrared Imager (LI-COR Biosciences).

### 2.9 Immunoaffinity purification

Standard protocols (Tomomori-Sato et al., 2013) were adapted to perform IP of SUMOylated proteins. Cells were lysed in 150 mM NaCl; 10 mM Tris pH 7.4; 1% Triton X-100, protease inhibitors (1 μg/mL aprotinin, 0.5 μg/mL leupeptine, 17.4 μg/mL PMSF) and 20 mM NEM when required. The lysate was sonicated for 4 s with a sonicator probe at power level 60 (Sonopuls, Bandelin). The lysate was then ultracentrifuged at 106,000 x *g* for 30 min at 4°C. Aliquots of the supernatant were taken (Input), and the remaining sample was incubated for 4 h at 4°C with anti-HA (Sigma) or anti-c-Myc (Sigma) agarose beads. The beads were washed twice in lysis buffer and proteins were eluted in Laemmli buffer (50 mM Tris pH 6.8; 10% glycerol; 0.2 g SDS; bromophenol blue; 33 mM DTT). Eluates (1/3 of the IP eluate/lane) were analyzed by SDS-PAGE and WB. For IP of the APC/C, NEM was excluded from lysates, as it caused non-specific attachment of the APC/C to beads, two ultracentrifuge steps were performed before IP, and the lysate was transferred to a new chilled tube after each centrifugation, and after incubation of the lysate with beads, the beads were washed 4 times with lysis buffer. Anti-HA (Sigma) and anti-c-Myc (Sigma) agarose beads were used for the IP of the APC/C activators. Protein G Sepharose beads (GE Healthcare) were used with anti-APC3 antibody or an IgG isotype control (Jackson Immuno Research) for the IP of endogenous APC/C.

### 2.10 Subcellular fractionation

Subcellular fractions of adult mouse cortex were prepared according to a published protocol (Carlin et al., 1980), with slight modifications. All steps were performed at 4°C, and all solutions contained protease inhibitors (1 mg/mL aprotinin; 0.5 mg/mL leupeptine; 17.4 mg/mL PMSF). The Homogenate (H) was obtained by homogenizing the cortex in Solution A (0.32 M Sucrose; 1 mM HEPES pH 7.4; 1 mM MgCl_2_; 0.5 mM CaCl_2_) with a Dounce homogenizer (12 strokes; 900 rpm). The sample was centrifuged for 10 min at 1,400 x *g*. The supernatant was collected (synaptosomes, cytosol, mitochondria, and organelles; S1) and the pellet was resuspended in Solution A (nuclei; P1). The S1 fraction was centrifuged for 10 min at 13,800 x *g*, and the supernatant was collected (cytosol, microsomes; S2). To generate the P2 (mitochondria and crude synaptosome) fraction, the pellet from the second centrifugation was resuspended in Solution B (0.32 M Sucrose; 1 mM HEPES pH 7.4) and homogenized with a Dounce homogenizer (4 strokes; 900 rpm). Next, 2 mL of the P2 fraction were added to the top of a sucrose step gradient and centrifuged at 82,500 x *g*. The gradient was created using 4 mL 1.2 M Sucrose, 3 mL 1 M Sucrose; 3 mL 0.85 M Sucrose in layers (1 mM HEPES pH 7.4 in all layers). After centrifugation, the turbid layer between the 1 M and 1.2 M sucrose phases was collected (synaptosomes; Syn). To generate the crude post-synaptic density (PSD) fraction, Solution C (0.32 M Sucrose; 1% Triton X-100; 12 mM Tris, pH 8.1) was added to the collected interphase and incubated with mild shaking for 15 min before centrifugation at 32,000 x *g* for 20 min. The pellet was resuspended in a 1:1 mixture of Solution B and Solution C. After 5 min, the pellet was resuspended by pipetting. One-third of the PSD fraction was loaded and 20 μg of protein from the remaining fractions were loaded per well for SDS-PAGE. Subcellular fractionation of HEK293FT cells was performed following the manufacturer’s protocol, but the nuclear pellet was washed with ice-cold PBS to increase the purity (NE-PER; Thermo Scientific). All buffers contained protease inhibitors (1 mg/mL aprotinin; 0.5 mg/mL leupeptine; 17.4 mg/mL PMSF) and NEM (20 mM, Sigma #E1271-5 g). The amount of protein per fraction analyzed by SDS-PAGE was identical in mass for the Equal Protein Loading samples (E) or equal in the percent of the cell volume in the Cell Equivalent samples (P). For data analysis, the experimental average of SUMOylated protein in the nucleus was subtracted from the ratio in the cytosol and this number was compared for all three experiments to a predicted value of 0 using a paired sample *t*-test in Excel (Supplementary Figure S5C).

### 2.11 Estimating APC4 turnover

APC4 levels were normalized to β-Tubulin levels, and the normalized-APC4 levels upon Cre NLS RFP-infection were then normalized to the normalized-APC4 levels upon control NLS RFP-infection. The resulting values were plotted and fit by an exponential curve, which was used to calculate the half-life of APC4 using standard procedures (Belle et al., 2006).

### 2.12 Fluorescence microscopy and image analysis

All images were acquired using a 63x oil-immersion objective on a Leica TCS SP2 confocal microscope (1,024 × 1,024 format size; 1.2 zoom; 193.74 nm x 193.74 nm size; 12-bit; 4-lines average). Blinding was achieved for image acquisition and analysis by coverslip coding. During acquisition, all conditions were imaged each day, and the microscope settings were stored to allow for the use of the same settings over several days. Images are displayed as collected, except for MAP2 (color levels changed to 3,080 with Fiji; rescue experiment). When a neuron did not fit into a single field of view, overlapping images were acquired and stitched (Fiji, Pairwise Stitching of Images; Linear Blending Method; check peaks 5; compute overlap; Preibisch et al., 2009; Schindelin et al., 2012). Z-stacks for β III-Tubulin and MAP2 signals were taken using a 92–108 nm step-size (4–15 slices/neuron), and a single maximum intensity projection was created to analyze β III-Tubulin. Scaled images were manually traced with the Fiji SNT plugin (Tavares et al., 2017; Hessian-based analysis: σ = 0.484 and max = 3.69). All neurites shorter than 3 μm were excluded from the analysis. For rescue experiments, neurites were only traced when they might be too short for analysis or the morphology was complex. Sholl Analysis was done using a 5 μm step size (3.1.110 plugin). For synapse counting, single plane images were taken of PSD95, RFP, Synapsin1/2, and MAP2 signals. Z-stacks were taken using a 92–108 nm step size (4–15 slices/neuron) for PSD95, Synapsin1/2, and MAP2. Scaled maximum projection images were used to determine total dendritic morphology (MAP2) with the Fiji NeuronJ plug-in (Meijering et al., 2004). Synapse numbers were determined with the Fiji SynapCountJ v2 plugin (Mata et al., 2017), which determines the number of synapses by quantifying co-localized PSD95 (threshold 120) and Synapsin1/2 (threshold 255) puncta within 1.94 μm from the traced MAP2 stain.

### 2.13 Statistics

While one experiment was analyzed by a paired *t*-test using Excel (Supplementary Figure S5C), the statistical analyses of biochemical experiments was done in Excel using an independent sample *t*-test. Imaging data for synapse quantification and neuron morphology did not have a Gaussian distribution and had unequal variance between samples, complicating the analysis. As our data fit a heavy-tailed distribution with unequal variance, we chose to use a Welch’s *t*-test instead of a non-parametric test, since the former is the most accurate test for such data (Skovlund and Fenstad, 2001; Fagerland and Sandvik, 2009; Kroeger et al., 2021). All statistical analyses of imaging data were performed using SPSS (IBM, version 27). The column graphs that include data from individual experiments were generated in Excel or in Prism 10.

## 3 Results

### 3.1 The neuronal APC/C contains APC4

Data obtained by depleting the APC/C activators, Cdh1 or Cdc20, have been the basis for implicating the APC/C in ubiquitylating a variety of neuronal substrates and in controlling neuron physiology (Eguren et al., 2011). As Cdh1 and Cdc20 can operate independently of the APC/C (Wan et al., 2011, 2017; Lee et al., 2015; Liu et al., 2016; Han et al., 2019) corresponding studies cannot unequivocally link observed effects to core APC/C dysfunction. Thus, it is not surprising that the levels of some alleged APC/C substrates are unaltered after APC2 KO (Kuczera et al., 2011). In view of these considerations, we attempted to assess APC/C function in neurons by depleting the core APC/C component APC4.

**Figure 1:**
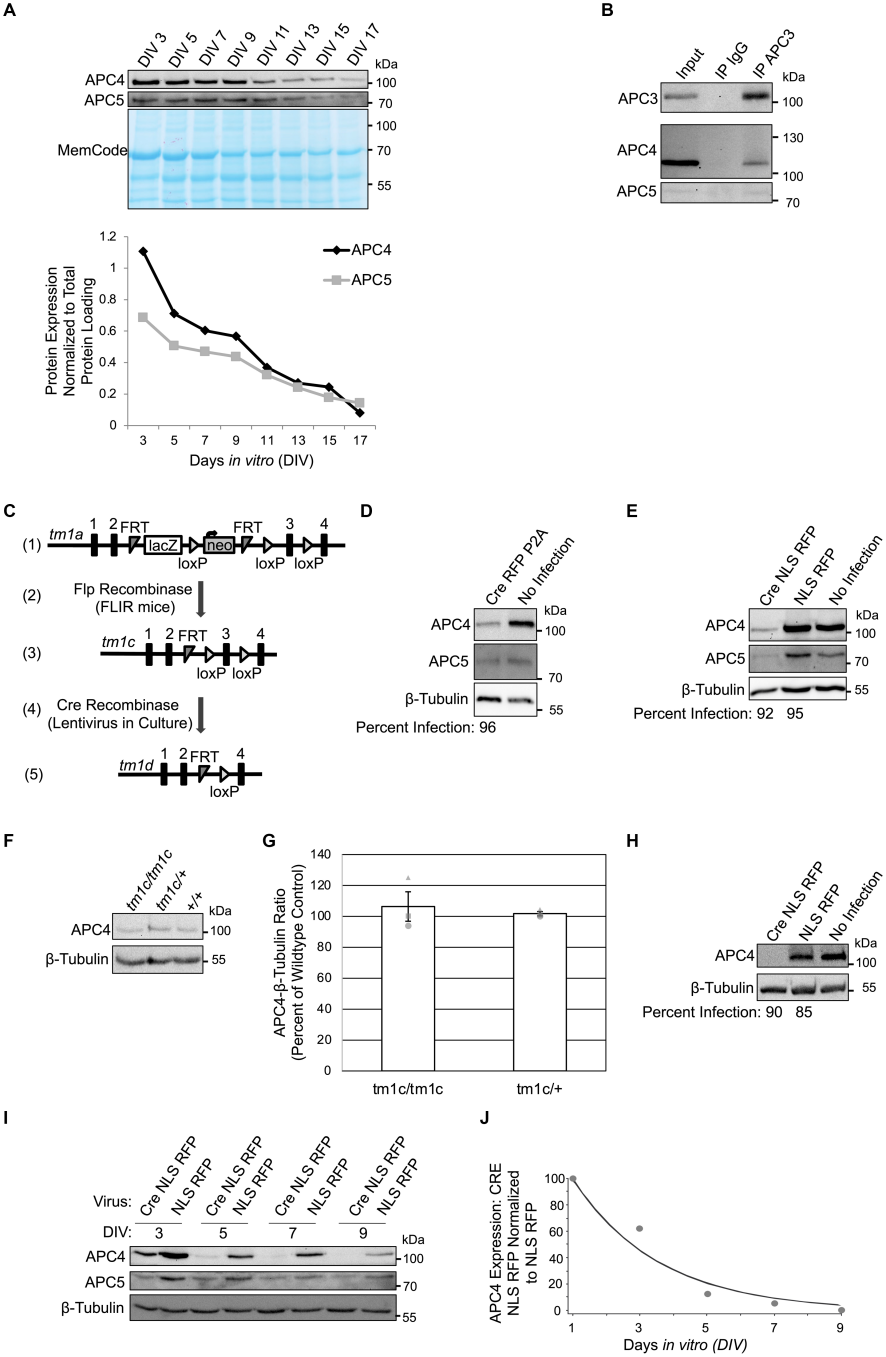
APC4 is an APC/C component in cortical neurons and APC4 is depleted from *ANAPC4* KO neurons. **(A)** The top panel shows WB analysis of APC4, APC5, and the total protein MemCode stain in wildtype primary cortical neuron cultures harvested at the indicated DIV. The bottom panel shows a graph depicting the quantification of the blots in the top panel after APC4 (black) and APC5 (gray) levels were normalized to MemCode. **(B)** WB analysis of APC3, APC4, and APC5 in wildtype DIV10 cortical cultures after lysis and IP with antibodies against APC3 or an IgG control (representative experiment). **(C)** The displayed strategy was used to generate the *ANAPC4 cKO mouse* allele. The *tm1a* allele has an insertion upstream of exon 3 of *ANAPC4* that contains *lacZ* and neomycin (neo) sequences, flanked by *FRT* and *loxP* sites (1). Mice with the *tm1a* allele were crossed to FLIR mice expressing Flp recombinase (2), generating the cKO *tm1c* allele by removing the cassette at the *FRT* sites (3). Neuron cultures generated from *ANAPC4 tm1c/tm1c* mice were infected with a lentivirus expressing Cre (4), which removes exon 3 and causes a frame shift that adds a premature stop codon. The resulting neurons express the *tm1d* allele, leading to the loss of APC4 (5). **(D)** WB shows the protein levels of APC4, APC5, and β-Tubulin in DIV11 primary hippocampal cultures prepared from *ANAPC4 cKO* neurons infected at DIV1 with Cre RFP P2A or a No Infection control. The percentage of infected cells (below WB panel) was determined by quantifying MAP2-positive cells co-expressing RFP. In Cre RFP P2A-infected cells, APC4 protein levels were reduced to ~28% and APC5 was reduced to ~62% of No Infection levels. **(E)** WB shows APC4, APC5, and β-Tubulin in DIV11 primary hippocampal cultures prepared from *ANAPC4 cKO* neurons infected at DIV1 with Cre NLS RFP, NLS RFP, or a No Infection control. The percentage of infected cells (below WB panel) was determined by quantifying MAP2-positive cells that co-express RFP. In Cre NLS RFP-infected cells, the levels of APC4 were reduced to ~16% and APC5 to ~35% of the levels in NLS RFP-infected cells. **(F)** Lysates of DIV10 cortical cultures obtained from mice with the *ANAPC4* alleles *tm1c/tm1c*, *tm1c/+*, or *+/+* were analyzed by WB with antibodies against APC4 and β-Tubulin (representative experiment). **(G)** Bar graph depicting the average APC4 levels normalized to β-Tubulin for the lysates in F. Error bars: standard error of the mean (SEM) for three experiments. Experiments 1, 2, and 3 are represented by a circle, triangle, and square, respectively. **(H–J)** Primary cortical cultures prepared from *ANAPC4 cKO* mice infected at DIV1 with Cre NLS RFP or NLS RFP were harvested in a large experiment at the indicated times. **(H)** WB shows APC4 and β-Tubulin in DIV11 cultures. The percentage of infected cells (below WB panel) was determined by quantifying MAP2-positive cells co-expressing RFP. APC4 was not detectable in Cre NLS RFP-infected cells. **(I)** WB shows APC4, APC5, and β-Tubulin levels over time. **(J)** Line graph depicting normalized APC4 levels from I. APC4 levels were first normalized to the β-Tubulin signal and then to normalized NLS RFP protein levels. The values in the line graph were fit by an exponential curve to determine APC4’s half-life (τ = 2.6 days, t_1/2_ = 1.8 days).

To confirm that neurons express APC4, we analyzed APC4 and APC5 protein levels in wildtype cortical cultures every 2 days *in vitro* (DIV). APC4 and APC5 levels decreased progressively from DIV3 but remained detectable until DIV17 (Figure 1A). To determine if APC4 is an APC/C component in neurons, we conducted APC3 IP and co-IP of APC4 and APC5. We found that APC4 and APC5 robustly and specifically associate with APC3 in neurons, indicating that APC4 is a component of the neuronal APC/C (Figure 1B).

### 3.2 Neuronal APC4 deletion affects APC5 stability

**Figure 2:**
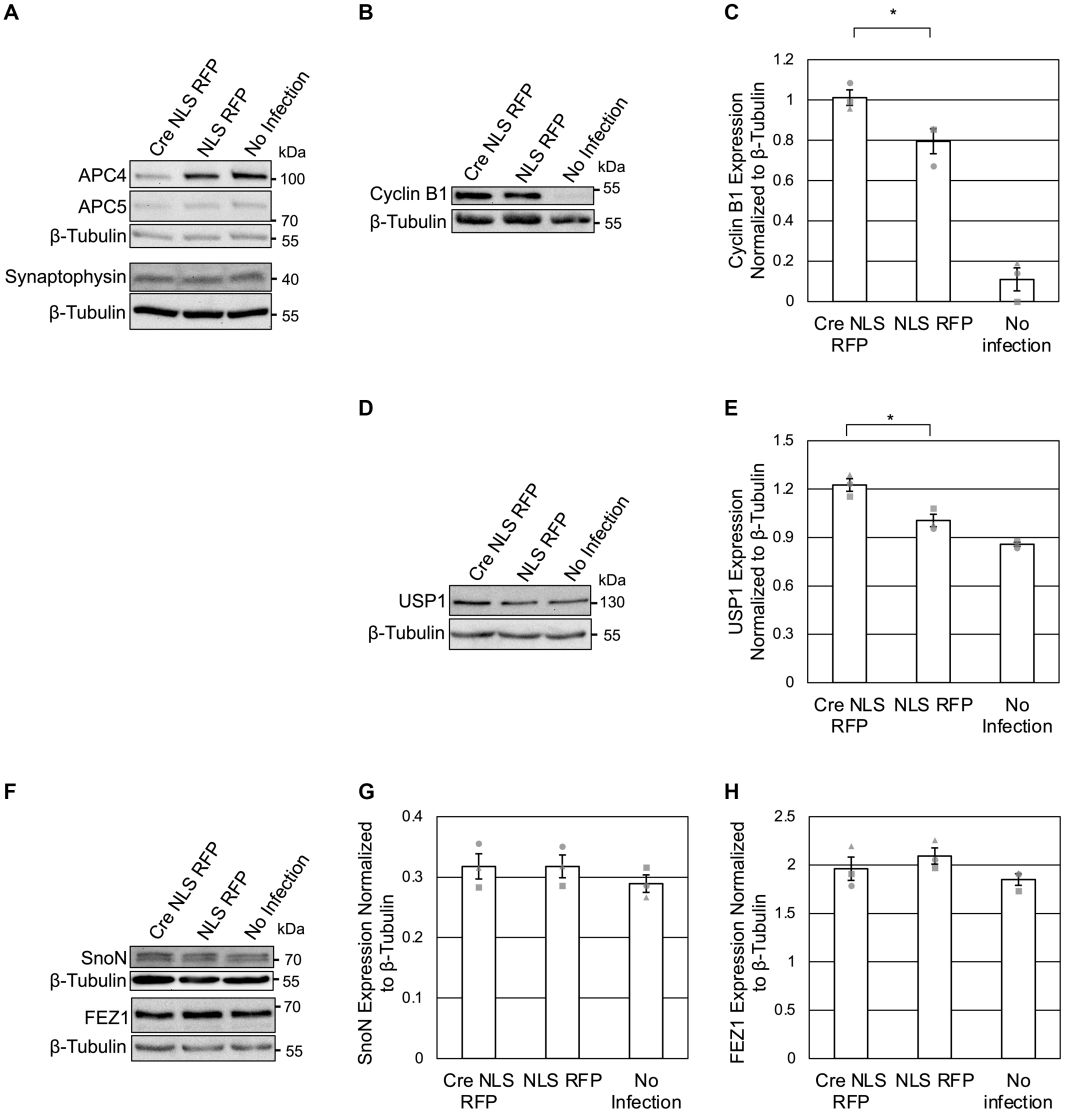
APC4 loss is accompanied by USP1 accumulation in DIV5 neurons. **(A–H)** Cortical neurons were prepared from *ANAPC4* cKO mice, and infected at DIV1 with Cre NLS RFP-or NLS RFP-expressing lentivirus. All cultures were harvested at DIV5, and lysates were analyzed by WB. Protein quantification was done by averaging values for three independent experiments (representative experiment displayed). Experiments 1, 2, and 3 are represented by a circle, triangle, and square, respectively. Error bars: SEM. **(A)** WB shows APC4, APC5, Synaptophysin, and β-Tubulin. **(B)** WB shows Cyclin B1 and β-Tubulin. **(C)** Bar graph depicting the average Cyclin B1 protein levels normalized to β-Tubulin. Cyclin B1 protein levels were increased in Cre NLS RFP-infected samples (asterisk: significance; t(2) = 2.971, *p* = 0.041). **(D)** WB shows USP1 and β-Tubulin. **(E)** Bar graph depicting the average levels of USP1 normalized to β-Tubulin. USP1 protein was increased in Cre NLS RFP-infected samples (asterisk: significance; t(2) = 4.008, *p* = 0.016). **(F)** WB shows SnoN, FEZ, and β-Tubulin. **(G)** Bar graph depicting the average levels of SnoN normalized to β-Tubulin. There was no significant difference in SnoN levels between Cre-and control-infected samples (*t*(2) = −0.001, *p* = 1.000). **(H)** Bar graph depicting FEZ1 normalized to β-Tubulin. There was no significant difference in FEZ1 levels between Cre-and control-infected samples (*t*(2) = −0.894, *p* = 0.422).

**Figure 3:**
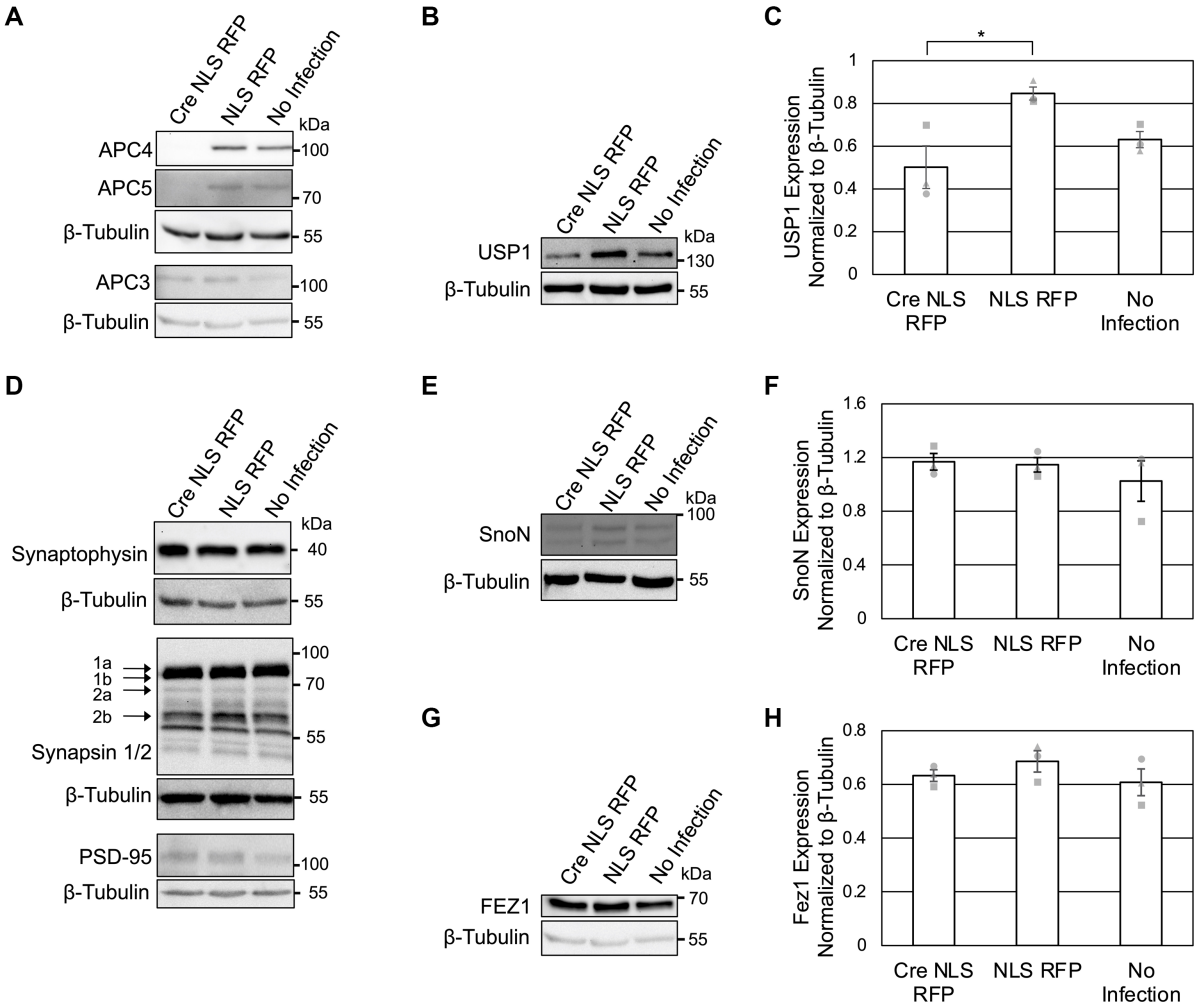
APC4 loss is accompanied by decreased USP1 protein levels in DIV11 neurons. **(A–H)** Cortical neurons were prepared from *ANAPC4* cKO mice and infected at DIV1 with Cre NLS RFP-or NLS RFP-expressing lentivirus. Cultures were harvested at DIV11, and lysates were analyzed by WB. Protein quantification was conducted by averaging values of three independent experiments (representative experiments displayed). Experiments 1, 2, and 3 are represented by a circle, triangle, and square, respectively. Error bars: SEM. **(A)** WB shows APC4, APC5, β-Tubulin, and APC3. **(B)** WB shows USP1 and β-Tubulin. **(C)** Bar graph depicting the average USP1 levels normalized to β-Tubulin. USP1 levels were increased in Cre NLS RFP-infected samples (asterisk: significance; t(2) = −3.299, *p* = 0.030). **(D)** WB shows Synaptophysin, β-Tubulin, Synapsin1/2, and PSD95. **(E)** WB shows SnoN and β-Tubulin. **(F)** Bar graph depicting the average levels of SnoN normalized to β-Tubulin. There was no significant difference in SnoN levels between Cre-and control-infected samples (*t*(2) = 0.274, *p* = 0.798). **(G)** WB shows FEZ1 and β-Tubulin. **(H)** Bar graph depicts the average levels of FEZ1 normalized to β-Tubulin. There was no significant difference in the FEZ1 levels between Cre-and control-infected samples (*t*(2) = −1.163, *p* = 0.155).

To stringently explore the function of the APC/C in neurons, we genetically deleted an essential APC/C core component, rather than an activator. We obtained an *ANAPC4* cKO mouse (IMPC, n.d.; Figure 1C-3). Cultured neurons were generated from cKO mice (*tm1c/tm1c*) and infected with a Cre-expressing lentivirus at DIV1, generating *ANAPC4* KO cells (*tm1d/tm1d*; Figure 1C). To assess the efficacy of APC4 depletion, we analyzed APC4 expression in lysates of infected cultures. We found that APC4 was depleted by DIV11 when hippocampal neuron cultures were infected with Cre RFP P2A- (Figure 1D) or Cre NLS RFP-lentivirus (Figure 1E).

In control experiments, we determined that the cKO allele, *tm1c,* does not affect endogenous APC4 expression. Mice were crossed to generate wild-type (*+/+*), heterozygous (*tm1c/+*), or homozygous (*tm1c/tm1c*) offspring. Lysates of cortical DIV11 neurons cultured from these mice exhibited comparable endogenous APC4 expression (Figures 1F,G).

We next determined the time course of APC4 loss upon Cre-infection in *ANAPC4* cKO cortical neurons. APC4 levels in KO neurons decayed rapidly to ~25% of wildtype levels by DIV5, almost completely by DIV9, and completely by DIV11. The decay was exponential, with τ = 2.6 days and t_1/2_ = 1.8 days (Figures 1H–J).

Human cytomegalovirus viral infection targets APC4, APC5, and APC1 for degradation, resulting in loss of APC/C activity. Knockdown of any one of these components was shown to cause depletion of all three components (Wiebusch et al., 2005; Thornton et al., 2006; Tran et al., 2010; Clark and Spector, 2015). We also found that APC5 expression is drastically reduced upon APC4 depletion (Figures 1D,E,I). Interestingly, APC4 depletion was more pronounced in cortical neurons (Figure 1H) than in hippocampal neurons (Figure 1E), and APC5 expression was more strongly co-depleted in cortical neurons (Figures 1I, 3A). The parallel loss of APC5 upon APC4 depletion implies that APC/C function is compromised in *ANAPC4* KO neurons. We subsequently used cortical neurons in experiments, because APC4 depletion is more pronounced in these cells.

### 3.3 The neuronal APC/C regulates USP1

Since prior studies implicated Cdh1 and Cdc20 in the ubiquitylation and degradation of several neuronal substrates (Eguren et al., 2011), we tested whether these effects require the APC/C by analyzing the levels of previously-published substrates at DIV5 (Figure 2) and DIV11 (Figure 3) in *ANAPC4* KO cortical neurons.

As APC4 and APC5 are both depleted under our conditions (Figures 2A, 3A), the APC/C is likely inactive. To support this notion, we first assessed the protein levels of Cyclin B1, a canonical APC/C substrate involved in maintaining neurons in G0 (Almeida et al., 2005; Maestre et al., 2008; Malureanu et al., 2010; Ledvin et al., 2023). As expected, Cyclin B1 levels were increased upon APC4 loss at DIV5 (Figures 2B,C). Since Cyclin B1 is not detectable in neuron cultures after DIV7 (Almeida et al., 2005), we did not analyze Cyclin B1 at DIV11. Our findings indicate that the *ANAPC4* KO system is well suited to study the effects of APC/C inactivation on putative substrates and to screen for novel substrates and phenotypes.

To possibly identify novel phenotypes associated with neuronal APC/C dysfunction, we analyzed the levels of a selected subset of neuronal markers at DIV 5 (Figure 2A) and DIV11 (Figure 3D). We did not detect alterations in the levels of Synaptophysin (Figures 2A, 3D), Synapsins (Figure 3D), or PSD95 (Figure 3D) in KO neurons, indicating that the APC/C does not regulate these proteins.

The deubiquitinating enzyme, USP1, was suggested to be a substrate of Cdh1-APC/C in cycling cells (Cotto-Rios et al., 2011; Cataldo et al., 2013). Its substrate status in neurons is unknown, but it is expressed in neurons and thought to regulating neuron morphology (Anckar and Bonni, 2015). We analyzed USP1 in *ANAPC4* KO cultures and detected increased USP1 levels in DIV5 KO neurons (Figures 2D,E), indicating that USP1 may be an APC/C substrate in cortical neurons. However, USP1 protein levels were decreased in *ANAPC4* KO cells at DIV 11 (Figures 3B,C), indicating that an as yet unidentified APC/C substrate either directly or indirectly regulates USP1 in DIV11 cortical neurons.

We next analyzed previously-published candidate APC/C substrates to determine if their degradation requires the neuronal APC/C and not just Cdh1 or Cdc20. Specifically, we tested *ANAPC4* KO lysates to determine if the levels of SnoN, an axon-growth-inducing transcriptional regulator proposed to be regulated by Cdh1-APC/C (Ikeuchi et al., 2009) and the levels of FEZ1, a protein involved in neurite development and intracellular transport and is proposed to be regulated by Cdc20-APC/C (Watanabe et al., 2014), were altered. Strikingly our data are consistent with prior data from APC2 KO neurons (Kuczera et al., 2011), as we also found that the levels of SnoN and FEZ1 are unaltered in DIV5 (Figures 2F–H) and DIV11 (Figures 3E–H) *ANAPC4* KO neurons.

### 3.4 The APC/C does not regulate synaptogenesis

Studies employing Cdc20 knockdown in neurons led to the notion that a pathway involving APC/C-mediated NEUROD2 ubiquitylation and downstream regulation of Complexin 2 plays a key role in synaptogenesis (Yang et al., 2009). However, two lines of evidence challenge this notion. First, there are no changes in NEUROD2 expression or synapse numbers in mutant mice with decreased Cdc20 (Malureanu et al., 2010). Second, the complete deletion of all Complexin paralogues also has no effect on synapse numbers (Reim et al., 2001; López-Murcia et al., 2019). In view of these discrepancies, we tested whether APC4 deletion itself affects synaptogenesis or alters NEUROD2 and Complexins levels.

We first assessed Complexin levels upon *ANAPC4* KO in cortical neurons at DIV11, when Complexin expression peaks (Reim et al., 2005), and found no changes in Complexin 1, Complexin 2, or Complexin 3 levels (Supplementary Figures S1A–D). While Complexin 3 is normally not detectable in neuron cultures, it becomes detectable upon viral infection (Supplementary Figure S1D). We next analyzed the levels of NEUROD2 in KO lysates, and again did not detect alterations at DIV5 or DIV11 (Supplementary Figures S1E,F). These data show that the APC/C does not regulate NEUROD2 or Complexins in cultured cortical neurons.

While we found that the APC/C does not modulate NEUROD2 or Complexin levels in cortical neurons, it could still employ another mechanism to regulate synaptogenesis. Therefore, we counted synapse numbers in *ANAPC4* KO neurons by quantifying the number of co-localized Synapsin1/2 and PSD95 puncta (Supplementary Figure S1G), which were the markers used in the original study (Yang et al., 2009). Synapse numbers were not altered in KO neurons (Supplementary Figure S1H), indicating that the APC/C does not regulate synaptogenesis in DIV11 cortical neurons.

### 3.5 The APC/C regulates primary neurite formation

**Figure 4:**
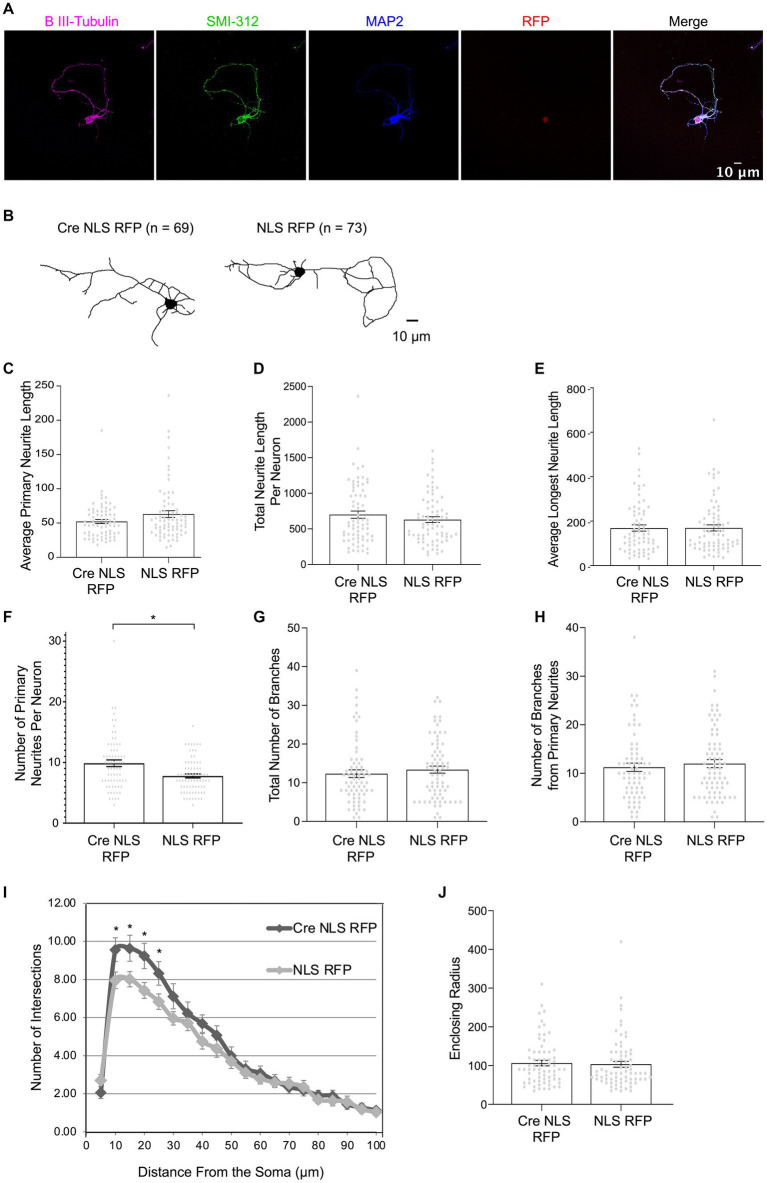
APC4-deficient cortical neurons form a greater number of primary neurites **(A–J)** Primary cortical neurons were prepared from *ANAPC4* cKO mice, and Cre-or control-infected neurons were fixed at DIV5, immunolabeled, and imaged. The β III-Tubulin label was traced and neuron morphology was then analyzed from these traces. Statistical analysis compared the populations of Cre NLS RFP- (*n* = 69) and NLS RFP-infected neurons (*n* = 73). Asterisks: significant differences; error bars: SEM; circles: individual data points. **(A)** Representative images of a neuron infected with Cre NLS RFP shows immunolabeling of β III-Tubulin (magenta), SMI-312 (green), MAP2 (blue), RFP (red), and the merged images (black). Overlapping MAP2 and SMI-312 patterns indicate that the axon and dendrites are not specified by DIV5. Scale bar: 10 μm. **(B)** Representative skeletonized traces of β III-Tubulin in Cre NLS RFP- (left) and NLS RFP-infected neurons (right). Scale bar: 10 μm. **(C)** Bar graph depicts the average primary neurite length/cell (*t*(120.829) = −1.919, *p* = 0.057). **(D)** Bar graph depicts the average total neurite length, which is the sum of the lengths of all primary, secondary, and tertiary neurites (*t*(130.812) = 1.083, *p* = 0.281). **(E)** Bar graph depicts the average length of the longest neurite (*t*(139.700) = −0.021, *p* = 0.984). **(F)** Bar graph depicts the average number of primary neurites ≥3 μm (*t*(111.91) = 3.251, *p* = 0.002). **(G)** Bar graph depicts the average of the total number of neurite branches per cell (*t*(138.785) = −0.661, *p* = 0.510). **(H)** Bar graph depicts the average number of branches that form off a primary neurite. **(I)** Graph depicts Sholl analysis of traced neurons (10 μm: t(122.207) = 2.119, *p* = 0.036; 15 μm: t(110.252) = 2.051, *p* = 0.043; 20 μm: t(114.993) = 2.311, *p* = 0.023; 25 μm: t(118.033) = 2.002, *p* = 0.048; 30 μm: t(103.932) = 1.546, *p* = 0.125). **(J)** Bar graph depicts the average enclosing radius of a neuron (*t*(139.496) = 0.283, *p* = 0.777).

Cdc20 and Cdh1 knockdown studies implicated the APC/C in ubiquitylating a variety of different substrates, which, in turn, were proposed to regulate neuron morphology, including neurite length and complexity (Bobo-Jiménez et al., 2017), axon length (Konishi et al., 2004; Lasorella et al., 2006; Stegmüller et al., 2006; Kannan et al., 2012), and dendrite length and complexity (Kim et al., 2009; Watanabe et al., 2014). However, these morphological phenotypes have usually not been formally linked to core APC/C components, which is problematic because Cdc20 and Cdh1 have APC/C-independent functions. To determine if the APC/C indeed regulates neuronal morphology, we analyzed neurite length and complexity in cortical *ANAPC4* KO neurons at DIV5, when neurite morphology can easily be assessed and APC4 expression is heavily depleted (Figures 1I,J). Similarly aged neurons (DIV3 to DIV5) were also used in earlier studies that addressed the role of the APC/C activators in neurons, with no major differences in effects between DIV3 and DIV5 neurons (Konishi et al., 2004; Lasorella et al., 2006; Stegmüller et al., 2006; Kim et al., 2009; Kannan et al., 2012; Watanabe et al., 2014). Like prior studies (Kempf et al., 1996), we could not reliably distinguish between dendrites and axons using antibodies against MAP2 and SMI-312 (Figure 4A), so we used β III-Tubulin immunolabeling to analyze all neurites longer than 3 μm (Figure 4B).

Prior studies indicated that Cdh1 knockdown increases axon length (Konishi et al., 2004; Lasorella et al., 2006; Stegmüller et al., 2006; Kannan et al., 2012) and that Cdc20 and APC2 knockdown decrease dendrite length (Kim et al., 2009; Watanabe et al., 2014). Hence, we expected to observe changes in dendrite length in *ANAPC4* KO neurons, but we did not detect any corresponding alterations (Figures 4C–E). Primary neurites were defined as neurites directly exiting the somata. Subsequent branch levels were labeled as secondary and tertiary depending on their origin. We compared the lengths of primary, secondary, and tertiary neurites individually (Figure 4C; secondary and tertiary data not shown), and the total length of all neurites (Figure 4D), but detected no KO-induced changes, indicating that the APC/C does not regulate neurite length in DIV5 cortical neurons. To assess possible changes in axon length, we measured the length of the longest neurite (Figure 4E) and detected no alterations.

While we were unable to confirm previously-proposed roles of the APC/C in regulating neurite length and complexity, we detected a novel phenotype. Cortical KO neurons had increased numbers of primary neurites exiting their somata (Figure 4F). As the average neurite length was not changed (Figure 4C), our data indicate that the APC/C regulates the initiation of neurite formation and not neurite stabilization or extension.

We next tested whether the APC/C regulates neurite complexity and branching, parameters that had been linked to Cdc20-APC/C (Kim et al., 2009). We quantified the total number of branches per cell (Figure 4G) and the total number of branches off primary neurites (Figure 4H), but did not detect effects of *ANAPC4* KO. We also analyzed the total neuron complexity with Sholl analysis by quantifying the number of times neurites intersect with equally-spaced concentric circles around the center of the neuron (Sholl, 1953; Tavares et al., 2017). We detected increased intersections at 10–25 μm distance from the center of the somata in APC4-deficient cells (Figure 4I), which can be explained by the increase in the number of primary neurites exiting the somata (Figure 4F). Finally, we did not observe alterations of the radius enclosing *ANAPC4* KO neurons (Figure 4J). These data indicate that the APC/C does not affect the size and complexity of cortical neurons at DIV5, but instead regulates the formation of neurites exiting somata.

### 3.6 SUMOylation of APC4 does not affect USP1 levels and neuronal morphology

Several proteomic studies (Matic et al., 2010; Schimmel et al., 2014; Cubeñas-Potts et al., 2015; Hendriks et al., 2018) as well as our own analyses of HA-His_6_-SUMO1 knock-in mice (Tirard et al., 2012; data not shown) identified APC4 as a SUMOylation target, and lysines 772 and 798 were shown to be SUMOylated in human APC4 (Eifler et al., 2018; Lee et al., 2018; Yatskevich et al., 2021). We mutated the corresponding lysines 772 and 797 in mouse APC4 to generate a Myc-APC4^K772R/K797R^ construct and showed that this variant cannot be SUMOylated (Supplementary Figure S2). Surprisingly, APC4 SUMOylation was very stable in the absence of NEM, an irreversible inhibitor of SENPs (Supplementary Figure S2B, arrow), indicating that these SUMOylated APC4 residues are inaccessible to SENPs.

We next tested if APC4 SUMOylation affects APC/C formation or the subcellular localization of APC4. We found that APC4 SUMOylation does not alter APC/C formation in HEK293 cells (Supplementary Figures S3A,B) or the binding of the APC/C to the APC/C activators, and that the complex primarily exists in a state where APC4 is not SUMOylated (Supplementary Figures 3A–D). We then examined whether APC4 SUMOylation affects APC4 localization. Despite major efforts to optimize fixation and immunolabeling protocols to visualize endogenous APC4 by confocal microscopy, the specific APC4 signal (absent in KO neurons) remained weak and was difficult to distinguish from background. Hence, we sought to biochemically assess the subcellular localization of SUMOylated APC4. We found that SUMOylation does not impact the gross subcellular localization of APC4, as HEK293 cells had equal fractions of Myc-APC4^WT^ and Myc-APC4^K772R/K797R^ in nuclear and cytoplasmic fractions (Supplementary Figure S4).

As SUMOylation of APC4 does not affect APC4 localization (Supplementary Figure S4), APC/C formation, or activator binding (Supplementary Figures S3A–D), we tested if APC4 SUMOylation affects APC/C function, as other studies had indicated (Eifler et al., 2018; Lee et al., 2018; Yatskevich et al., 2021). We first analyzed the subcellular localization of APC4 and SUMOylated APC4. Upon subcellular fractionation of mouse cortex, we detected APC4 in all fractions, including synaptosomes (Syn) and the crude PSD (PSD) fractions. APC4 was most abundant in the cytosolic fraction (S2), where it was strongly SUMOylated (Supplementary Figure S5A). In cycling HEK293 cells, APC4 was present in cytosolic and nuclear fractions, but SUMOylated APC4 was enriched in the nucleus (Supplementary Figures S5B,C).

**Figure 5:**
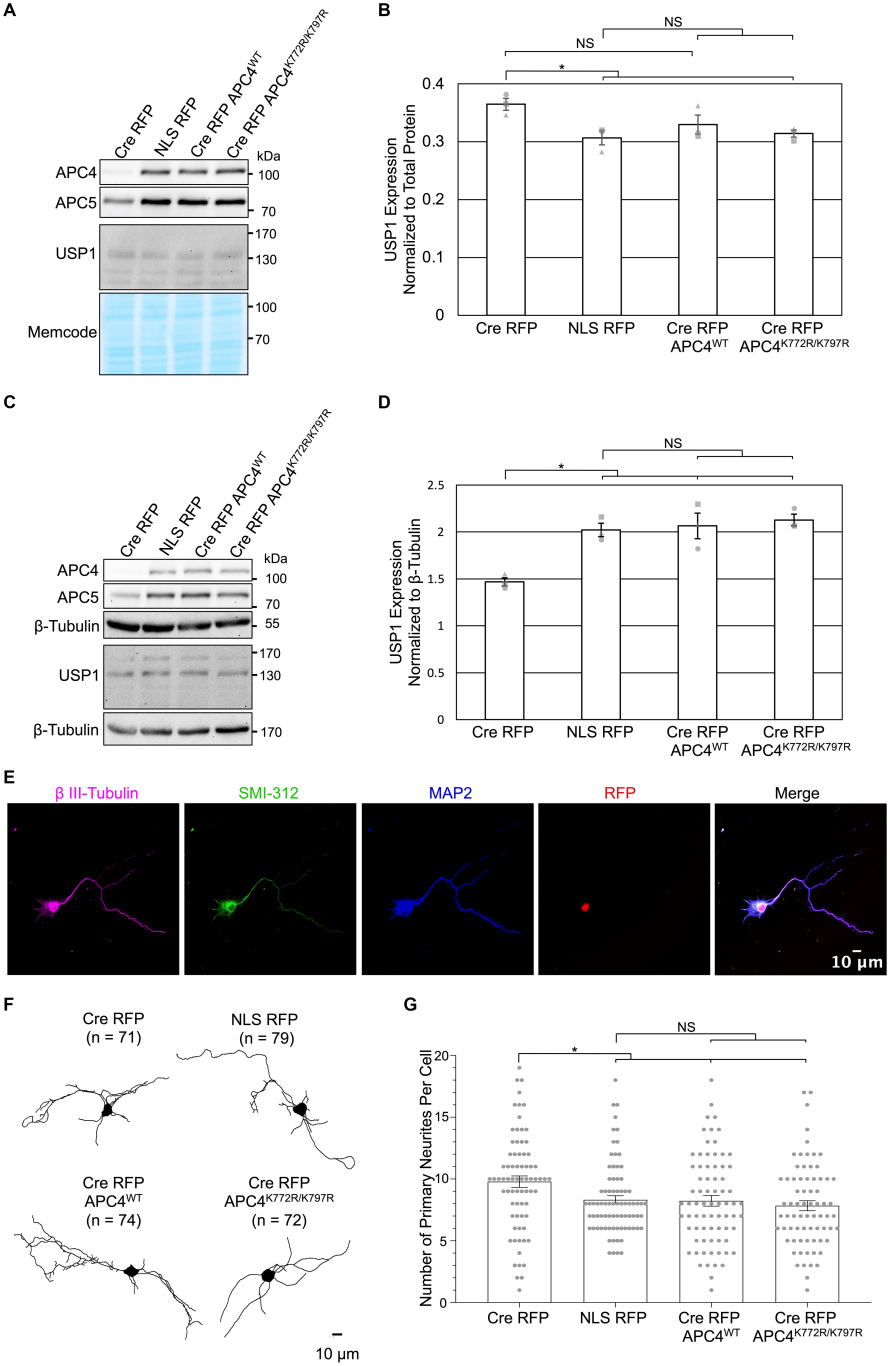
APC4 SUMOylation does not affect APC/C-dependent regulation of USP1 protein levels in DIV5 or DIV11 neurons. **(A–G)** Primary cortical neurons were prepared from *ANAPC4* cKO mice, infected at DIV1, and harvested at DIV5 or DIV11. Neurons were infected with lentivirus expressing Cre RFP, NLS RFP control, or Cre-expressing rescue constructs in the form of Cre RFP APC4^WT^ (wildtype) or Cre RFP APC4^K772R/K797R^ (SUMOylation-deficient). **(A)** WB analysis of DIV5 lysates immunoblotted for APC4, APC5, USP1, and MemCode (representative experiment). **(B)** Bar graph depicts average USP1 protein levels normalized to MemCode from three independent experiments. USP1 protein levels were elevated in Cre RFP-infected samples (*t*(2) = 3.605., *p* = 0.023), and USP1 protein levels were fully rescued to control levels by expressing APC4^K772R/K797R^ (SUMOylation-deficient; t(2) = 4.180, *p* = 0.014) and there was a trend of rescue when APC4^WT^ (wildtype; t(2) = 1.801, *p* = 0.146) was expressed. Experiments 1, 2, and 3 are represented by a circle, triangle, and square, respectively. Asterisk: significant difference; NS: no significant difference; error bars: SEM. **(C)** WB analysis of DIV11 lysates immunoblotted for APC4, APC5, USP1, and β-Tubulin (representative experiment). **(D)** Bar graph depicts average USP1 levels normalized to β-Tubulin from three independent experiments. USP1 was decreased in Cre RFP-infected samples (*t*(2) = −6.598, *p* = 0.003), and the expression of Cre RFP APC4^WT^ (wildtype; t(2) = −4.166, *p* = 0.014) and Cre RFP APC4^K772R/K797R^ (SUMOylation-deficient; t(2) = −8.756, *p* = 0.001) rescued this decrease. Experiments 1, 2, and 3 are represented by a circle, triangle, and square, respectively. Asterisk: significant difference; NS: no significant difference; error bars: SEM. **(E–G)** Maximum projection images of β III-tubulin was used to determine the number of neurites greater than 3 μm long that exit the somata on DIV5 cortical neurons. **(E)** Representative images of DIV5 neurons that were fixed, immunostained, and imaged. Scale bar: 10 μm. **(F)** Representative skeletonized traces of β III-Tubulin immunolabeling. Scale bar: 10 μm; (*n*), cells analyzed. **(G)** Bar graph depicts the average number of primary neurites longer than 10 μm. There was an increase in the number of primary neurites exiting the somata of neurons infected with Cre RFP (*t*(127.462) = 2.602, *p* = 0.010), and this phenotype was rescued by the expression of Cre RFP APC4^WT^ (wildtype; t(136.883) = 3.201, *p* = 0.002) and Cre RFP APC4^K772R/K797R^ (SUMOylation-deficient; t(117.113) = 6.964, *p* < 0.001). Asterisk: significant difference; NS: no significant difference; error bars: SEM; circles: individual data points.

We next examined if APC4 SUMOylation is involved in APC/C-dependent regulation of USP1 levels and the number of neurites exiting somata. We previously observed increased USP1 protein levels in DIV5 KO neurons (Figures 2D,E) and decreased USP1 protein levels in DIV11 KO neurons (Figures 3B,C). We repeated this analysis with cortical *ANAPC4* KO neurons infected with lentiviruses expressing Cre RFP, NLS RFP, or rescue viruses expressing Cre RFP APC4^WT^ (wildtype) or Cre RFP APC4^K772R/K797R^ (SUMOylation-deficient). The levels of APC4 and APC5 were fully rescued in Cre RFP APC4^WT^-and Cre RFP APC4^K772R/K797R^-infected cultures, indicating that APC/C integrity was rescued (Figures 5A–D). Consistent with our prior experiments, we saw increased USP1 levels at DIV5 (Figures 5A,B) and decreased levels at DIV 11 (Figures 5C,D) in KO cultures. These changes in USP1 levels were rescued in Cre RFP APC4^WT^-and Cre RFP APC4^K772R/K797R^-infected cultures (Figures 5A–D), indicating that USP1 is indeed regulated, directly or indirectly, by the APC/C, albeit independently of APC4 SUMOylation. In a final set of experiments, we tested if APC4 SUMOylation affects the ability of the APC/C to regulate the number of primary neurites exiting somata (Figure 4F). We imaged DIV5 cortical neurons and analyzed primary neurite numbers using β III-Tubulin immunolabeling (Figures 5E–G). Similar to what we had observed previously, *ANAPC4* KO caused an increase in the number of neurites exiting somata (Figure 5G), and this KO phenotype was fully rescued in Cre RFP APC4^WT^-and Cre RFP APC4^K772R/K797R^-infected cultures (Figure 5G). This indicates that the APC/C does indeed regulate the number of primary neurites exiting somata, albeit in a manner that does not require APC4 SUMOylation.

## 4 Discussion

The present study was designed to examine APC/C function in developing neurons. We shut down APC/C function in cortical neurons by conditional deletion of the core APC/C component, APC4. The phenotype of APC4-deficient neurons indicates that the APC/C regulates the number of primary neurites and differentially regulates USP1 protein levels at distinct developmental stages, and that these functions are independent of APC4 SUMOylation. Our data do not confirm prior studies employing depletion of the APC/C activators and indicating that the APC/C regulates neurite length and branching, synaptogenesis, or the levels of SnoN, NEUROD2, and FEZ1.

### 4.1 *ANAPC4* KO shuts down APC/C activity

After establishing that the neuronal APC/C contains APC4 (Figures 1A,B), we used a cKO mouse line to delete *ANAPC4*, the gene encoding APC4, in neuron cultures (Figure 1C). We calculated the APC4 protein half-life to be ~1.8 days in cortical cultures (Figure 1J), which is consistent with prior studies (Mathieson et al., 2018) and indicates that APC4 is rapidly depleted in cKO neurons upon Cre-mediated recombination.

Similar to studies on cycling cells (Thornton et al., 2006; Tran et al., 2010; Clark and Spector, 2015), we found that APC4 depletion causes the concomitant loss of APC5 in cortical (Figures 1I, 2A, 3A) and hippocampal (Figures 1D,E) neurons. This indicates that the loss of APC4 destabilizes the APC/C, rendering it dysfunctional. Indeed, studies on human cytomegalovirus show that the APC/C is not functional when APC4 or APC5 are depleted (Wiebusch et al., 2005; Tran et al., 2010). The fact that the canonical APC/C substrate, Cyclin B1, is increased upon *ANAPC4* KO (Figures 2B,C) supports the notion that *ANAPC4* KO leads to APC/C inactivation.

At first glance, the relatively modest increase in Cyclin B1 levels observed in KO neurons (Figures 2B,C) may appear surprising. In fact, this finding is not surprising for three reasons: (i) APC4 depletion is incomplete at DIV5 when we tested Cyclin B1 levels; (ii) the ability of the APC/C to ubiquitylate Cyclin B1 changes during the cell cycle and may vary in different cell types; (iii) other ubiquitin ligases, like Parkin, may control Cyclin B1 levels, which is also indicated by the fact that APC4 and Cyclin B1 levels decrease simultaneously during development (Lee et al., 2015).

### 4.2 APC/C inactivation and previously-proposed substrates

In our experiments, the levels of SnoN, FEZ1, and NEUROD2 were unaffected by *ANAPC4* KO neurons, indicating that these proteins are not APC/C substrates at DIV5 and DIV11 (Figures 2F–H, 3E–H; Supplementary Figures 1E,F). Our findings on SnoN are in accord with a previous study showing that APC2 KO in excitatory forebrain neurons does not affect SnoN levels, in spite of the fact that APC/C function requires APC2 (Wirth et al., 2004; Kuczera et al., 2011). While it is unclear why FEZ1 levels are unaffected in *ANAPC4* KO neurons, a possible explanation is that we worked with cultured neurons and the APC/C-FEZ1 link is based on an *in vivo* study (Watanabe et al., 2014).

One of the most intriguing APC/C substrates is NEUROD2, which was proposed to regulate synaptogenesis in an APC/C-modulated manner via a signaling pathway that involves Complexin 2 (Yang et al., 2009). We found NEUROD2 levels to be unaltered in *ANAPC4* KO neurons, indicating it is not an APC/C substrate in cortical neurons (Supplementary Figures S1E–F). In agreement with this, we did not detect changes in Complexin 1 and Complexin 2 levels upon APC4 loss (Supplementary Figures S1A–C). Complexin 3 levels are barely detectable in forebrain neuron cultures (Xue et al., 2008). Hence, it is intrinsically difficult to analyze Complexin 3 levels, and lentivirus infection increased Complexin 3 levels (Supplementary Figures S1A,D). Therefore, we are unable to make definitive statements regarding an interplay between the APC/C and Complexin 3. Similar, for instance, to human cytomegalovirus affecting the levels of APC/C components (Wiebusch et al., 2005; Thornton et al., 2006; Tran et al., 2010; Clark and Spector, 2015), lentiviral infection appears to affect the protein levels of Cyclin B1 (Figures 2B,C), Complexin 1 (Supplementary Figures S1A,B), and Complexin 3 (Supplementary Figures S1A,D).

Finally, we estimated the numbers of synapses per neuron in wildtype and KO cultures by counting PSD95 puncta co-localized with Synapsin1/2. While not strictly at single-synapse resolution, such an approach of combining co-labelling for pre-and postsynaptic markers and confocal imaging allows to estimate synapse numbers in neuron cultures with substantial reliability (Burgalossi et al., 2010, 2012), and a similar methodology was employed in the original study that indicated altered synapse density upon perturbation of Cdc20-APC/C function (Yang et al., 2009). Strikingly, we found no alterations in synapse numbers in KO neurons (Supplementary Figures S1G,H). Our data are in agreement with an earlier study employing a hypomorphic *Cdc20* deletion mutant mouse line, which is characterized by decreased Cdc20 expression but does not show altered NEUROD2 levels or changes in synapse numbers (Malureanu et al., 2010). Finally, a significant role of an APC/C-NEUROD2-Complexin pathway in synaptogenesis is in general implausible because Complexin-deficient neurons show no changes in synaptogenesis (Reim et al., 2001; López-Murcia et al., 2019).

### 4.3 APC/C inactivation and neuronal morphology

Multiple studies involving the perturbation of the APC/C activators, Cdc20 or Cdh1, implicated the APC/C in regulating neurite length and complexity (Bobo-Jiménez et al., 2017), dendrite length and complexity (Kim et al., 2009; Watanabe et al., 2014), and axon length (Lasorella et al., 2006; Stegmüller et al., 2006, 2008; Li et al., 2019). FEZ1 (Watanabe et al., 2014) and SnoN (Stegmüller et al., 2006, 2008; Li et al., 2019) were suggested to be APC/C substrates involved in this context. Our data do not support these findings. First, our data indicate that FEZ1 and SnoN are not APC/C substrates in cultured mouse forebrain neurons (Figures 2F–H, 3E–H). Further, we did not detect effects of *ANAPC4* KO on neurite length (Figures 4C–E) and branching (Figures 4G,H), or overall neurite complexity (Figures 4I,J). Likewise, axon morphology does not appear to be affected by *ANAPC4* KO, as the enclosing radii of neurons (Figure 4J) and the length of the longest neurite were unaltered (Figure 4E). In agreement with our data, a hypomorphic *Cdc20* deletion mutant mouse neurons also showed normal dendrite lengths (Malureanu et al., 2010).

### 4.4 APC/C regulation of USP1 and the number of primary neurites

USP1 removes ubiquitin from substrate proteins, thereby stabilizing the substrate. Similar to prior studies indicating that USP1 is a Cdh1-APC/C substrate in cycling cells (Cotto-Rios et al., 2011; Cataldo et al., 2013), we found that USP1 protein levels are upregulated in *ANAPC4* KO cortical cultures at DIV5 (Figures 2D,E). A prior study showed that the APC/C ubiquitylates USP1 *in vitro* (Cotto-Rios et al., 2011), but corresponding *in vitro* assays often lead to false positives, which is evident when one compares prior data on SnoN (Stegmüller et al., 2006, 2008; Li et al., 2019) with our data (Figures 2F,G, 3E,F). Hence, it is unclear whether the APC/C directly ubiquitylates USP1 in neurons, and methods to address this question are currently lacking.

Strikingly, elevated USP1 protein levels observed in KO cultures at DIV5 (Figures 2D,E) reverted to decreased levels at DIV11 (Figures 3B,C). This observation is compatible with the notion that the APC/C ubiquitylates an unknown substrate in DIV11 cortical neurons that either directly or indirectly regulates USP1 levels. The physiological relevance of this differential regulation of USP1 by the APC/C is unclear. While it might simply reflect a transient requirement for a certain functionality during neuronal differentiation or development, the phenomenon may also represent a feedback mechanism in neurons, where the cell senses elevated USP1 levels or altered USP1 substrate levels and responds by decreasing USP1 protein levels. Such a feedback mechanism would be important for cells with chronic USP1 overexpression, as USP1 regulates genomic stability, thereby affecting the ability of neurons to correct DNA damage. USP1 is currently a major target for the development of cancer therapeutics, as USP1 inhibitors are effective in treating cancers with a BRCA1 mutation (García-Santisteban et al., 2013; Simoneau et al., 2023). Neurodegeneration is also associated with a dysregulation of genomic stability and changes in APC/C substrates (Fuchsberger et al., 2016), so USP1 may also be dysregulated during neurodegeneration. Recent studies indicate that USP1 may also regulate the circadian clock (Hu et al., 2024). In view of these considerations, a detailed understanding of how the neuronal APC/C differentially regulates USP1 protein levels may be important for developing cancer and neurodegeneration treatments.

While we did not observe changes in neurite length (Figures 4C,E) or branching (Figures 4G,H) upon APC4 loss, we discovered a phenotype that had previously not been attributed to APC/C function, namely an increase in the number of neurites exiting the somata of cortical neurons (Figure 4F). Given that neurite lengths were not altered, our data indicate that the APC/C affects a very early step in neurite formation, likely the step where actin is rearranged to form new neurites (Flynn, 2013). Interestingly, USP1 depletion in cultured neurons was previously reported to induce a decrease in the number of primary neurites exiting the somata (Anckar and Bonni, 2015). We observed the opposite effect in *ANAPC4* KO neurons, where increased USP1 levels were correlated with more primary neurites (Figure 4F), so the APC/C may in fact regulate neuron morphology through a pathway that involves USP1. Unfortunately, elucidating this pathway and its involvement in regulating the number of primary neurites is complicated by a feedback mechanism connecting ID1, which is an USP1 substrate, to APC/C inhibition (Man et al., 2008; Chow et al., 2012).

### 4.5 APC4 SUMOylation does not impact APC/C activity

Our data confirm prior studies (Eifler et al., 2018; Lee et al., 2018) indicating that APC4 SUMOylation does not affect APC/C formation (Supplementary Figure S3) or APC4 localization (Supplementary Figure S4). Furthermore, we found that APC4 SUMOylation does not affect activator binding to the APC/C (Supplementary Figures 3C,D). Interestingly, the APC/C appeared to normally reside in a state where APC4 is non-SUMOylated, even when it was bound to an activator (Supplementary Figures 3C,D), which indicates that APC4 is only transiently SUMOylated. In view of this, we explored whether APC4 SUMOylation affects APC/C function.

We tested if APC4 SUMOylation affects the ability of the APC/C to regulate USP1 levels and neurite formation (Figures 2D,E, 3B,C, 4F). We found that wild-type and SUMOylation-deficient APC4 fully rescue the key *ANAPC4* KO phenotypes observed, including the concomitant loss of APC5, altered USP1 levels, and increased numbers of primary neurites. Our data indicate that these phenotypes are indeed due to *ANAPC4* KO and APC/C dysfunction, and that APC4 SUMOylation does not affect the corresponding APC/C functions (Figure 5).

Evidence from cycling cells indicates that APC4 SUMOylation affects the ability of APC/C to ubiquitylate a subset of substrates (Eifler et al., 2018; Lee et al., 2018; Yatskevich et al., 2021). For example, Hsl1 is ubiquitylated by the Cdh1-and the Cdc20-APC/C, but APC4 SUMOylation only affects the ability of the Cdh1-APC/C to ubiquitylate Hsl1 (Eifler et al., 2018; Yatskevich et al., 2021). The SUMOylated APC4 residues are located in the interior of the APC/C and thought to alter the conformation of the APC/C when it is bound to the MCC, which affects the ability of the Cdc20-APC/C to ubiquitylate Cyclin B1 and Securin (Yatskevich et al., 2021). Consistent with this notion, we show that APC4 SUMOylation is remarkably stable in buffers lacking NEM (Supplementary Figure S2B), indicating that the SUMOylated residues are not accessible to SENPs. While APC4 SUMOylation did not affect the ability of the APC/C to alter USP1 levels (Figures 5A–D), identification of novel neuronal APC/C substrates in the cytosol may enable the discovery of substrates that are likely differentially ubiquitylated by the SUMOylated complex.

### 4.6 Discrepancies between present and prior studies

Overall, our study indicates that many previously-proposed APC/C-linked substrates and phenotypes are not regulated by the APC/C in cortical neuron cultures. Several explanations can account for these discrepancies. First, species and cell-type differences may be the basis for the discrepant datasets, as we used mouse cortical neurons and other studies primarily employed rat cerebellar granule neurons. Second, knockdown approaches in neuron cultures are notorious for off-target effects that alter synaptic marker localization and neuronal morphology (Alvarez et al., 2006). Hence, some of the previously-published phenotypes may be due to off-target effects of corresponding knockdown approaches. Finally, most studies involved the depletion of Cdh1 or Cdc20 as a means to inactivate the APC/C, which is problematic because Cdh1 and Cdc20 can also function in an APC/C-independent manner (Wan et al., 2011, 2017; Kannan et al., 2012; Lee et al., 2015; Han et al., 2019). Hence, some of the substrates and phenotypes that were previously linked to the APC/C may be related to such “moonlighting” functions of Cdh1 and Cdc20. In any case, our data demonstrate that care must be taken when extrapolating APC/C function from experimental data obtained by perturbing APC/C activators, and that more stringent approaches are required to tie substrates and phenotypes to APC/C function.

## Data availability statement

The original contributions presented in the study are included in the article/Supplementary material, further inquiries can be directed to the corresponding author.

## Ethics statement

Ethical approval was not required for studies on humans in accordance with the local legislation and institutional requirements because only commercially available established cell lines were used. The animal study was approved by Niedersächsisches Landesamt für Verbraucherschutz und Lebensmittelsicherheit. The study was conducted in accordance with the local legislation and institutional requirements.

## Author contributions

JD: Conceptualization, Data curation, Formal analysis, Investigation, Methodology, Project administration, Resources, Validation, Visualization, Writing – original draft, Writing – review & editing. MT: Conceptualization, Funding acquisition, Supervision, Visualization, Writing – original draft, Writing – review & editing. NB: Conceptualization, Funding acquisition, Supervision, Visualization, Writing – original draft, Writing – review & editing.

## References

[ref1] Almeida A. Bolaños J. P. Moreno S. (2005). Cdh1/Hct1-APC is essential for the survival of postmitotic neurons. J. Neurosci. 25, 8115–8121. doi: 10.1523/jneurosci.1143-05.2005, PMID: 16148219 PMC6725543

[ref2] Alvarez V. A. Ridenour D. A. Sabatini B. L. (2006). Retraction of synapses and dendritic spines induced by off-target effects of RNA interference. J. Neurosci. 26, 7820–7825. doi: 10.1523/jneurosci.1957-06.2006, PMID: 16870727 PMC6674211

[ref3] Anckar J. Bonni A. (2015). Regulation of neuronal morphogenesis and positioning by ubiquitin-specific proteases in the cerebellum. PLoS One 10:e0117076. doi: 10.1371/journal.pone.0117076 25607801 PMC4301861

[ref4] Belle A. Tanay A. Bitincka L. Shamir R. O’Shea E. K. (2006). Quantification of protein half-lives in the budding yeast proteome. Proc. Natl. Acad. Sci. USA 103, 13004–13009. doi: 10.1073/pnas.0605420103, PMID: 16916930 PMC1550773

[ref5] Bobo-Jiménez V. Delgado-Esteban M. Angibaud J. Sánchez-Morán I. de la Fuente A. Yajeya J.. (2017). APC/CCdh1-Rock2 pathway controls dendritic integrity and memory. Proc. Natl. Acad. Sci. USA 114, 4513–4518. doi: 10.1073/pnas.1616024114 28396402 PMC5410848

[ref6] Burgalossi A. Jung S. Man K. N. Nair R. Jockusch W. J. Wojcik S. M.. (2012). Analysis of neurotransmitter release mechanisms by photolysis of caged Ca^2+^ in an autaptic neuron culture system. Nat. Protoc. 7, 1351–1365. doi: 10.1038/nprot.2012.074, PMID: 22722370

[ref7] Burgalossi A. Jung S. Meyer G. Jockusch W. J. Jahn O. Taschenberger H.. (2010). SNARE protein recycling by αSNAP and βSNAP supports synaptic vesicle priming. Neuron 68, 473–487. doi: 10.1016/j.neuron.2010.09.019, PMID: 21040848

[ref8] Carlin R. K. Grab D. J. Cohen R. S. Siekevitz P. (1980). Isolation and characterization of postsynaptic densities from various brain regions: enrichment of different types of postsynaptic densities. J. Cell Biol. 86, 831–845. doi: 10.1083/jcb.86.3.831, PMID: 7410481 PMC2110694

[ref9] Cataldo F. Peche L. Y. Klaric E. Brancolini C. Myers M. P. Demarchi F.. (2013). CAPNS1 regulates USP1 stability and maintenance of genome integrity. Mol. Cell. Biol. 33, 2485–2496. doi: 10.1128/mcb.01406-12, PMID: 23589330 PMC3700094

[ref10] Chen B.-J. Lam T. C. Liu L.-Q. To, C.-H (2017). Post-translational modifications and their applications in eye research. Mol. Med. Rep. 15, 3923–3935. doi: 10.3892/mmr.2017.6529, PMID: 28487982

[ref11] Chow C. Wong N. Pagano M. Lun S. Nakayama K. Nakayama K.. (2012). Regulation of APC/C Cdc20 activity by RASSF1A–APC/C Cdc20 circuitry. Oncogene 31, 1975–1987. doi: 10.1038/onc.2011.372, PMID: 21874044 PMC3325600

[ref12] Clark E. Spector D. H. (2015). Studies on the contribution of human cytomegalovirus UL21a and UL97 to viral growth and inactivation of the anaphase-promoting complex/Cyclosome (APC/C) E3 ubiquitin ligase reveal a unique cellular mechanism for downmodulation of the APC/C subunits APC1, APC4, and APC5. J. Virol. 89, 6928–6939. doi: 10.1128/jvi.00403-15, PMID: 25903336 PMC4468507

[ref13] Cotto-Rios X. M. Jones M. J. K. Busino L. Pagano M. Huang T. T. (2011). APC/CCdh1-dependent proteolysis of USP1 regulates the response to UV-mediated DNA damage. J. Cell Biol. 194, 177–186. doi: 10.1083/jcb.201101062, PMID: 21768287 PMC3144416

[ref14] Cubeñas-Potts C. Srikumar T. Lee C. Osula O. Subramonian D. Zhang X.. (2015). Identification of SUMO-2/3-modified proteins associated with mitotic chromosomes. Proteomics 15, 763–772. doi: 10.1002/pmic.201400400 25367092 PMC4445636

[ref15] Daniel J. A. Cooper B. H. Palvimo J. J. Zhang F.-P. Brose N. Tirard M. (2017). Analysis of SUMO1-conjugation at synapses. eLife 6:e26338. doi: 10.7554/elife.26338, PMID: 28598330 PMC5493437

[ref16] Eguren M. Manchado E. Malumbres M. (2011). Non-mitotic functions of the anaphase-promoting complex. Semin. Cell Dev. Biol. 22, 572–578. doi: 10.1016/j.semcdb.2011.03.010, PMID: 21439391

[ref17] Eifler K. Cuijpers S. A. G. Willemstein E. Raaijmakers J. A. Atmioui D. E. Ovaa H.. (2018). SUMO targets the APC/C to regulate transition from metaphase to anaphase. Nat. Commun. 9:1119. doi: 10.1038/s41467-018-03486-4, PMID: 29549242 PMC5856775

[ref18] Fagerland M. W. Sandvik L. (2009). Performance of five two-sample location tests for skewed distributions with unequal variances. Contemp. Clin. Trials 30, 490–496. doi: 10.1016/j.cct.2009.06.007, PMID: 19577012

[ref19] Flynn K. C. (2013). The cytoskeleton and neurite initiation. BioArchitecture 3, 86–109. doi: 10.4161/bioa.26259 24002528 PMC4201609

[ref20] Fuchsberger T. Martínez-Bellver S. Giraldo E. Teruel-Martí V. Lloret A. Viña J. (2016). Aβ induces excitotoxicity mediated by APC/C-Cdh1 depletion that can be prevented by glutaminase inhibition promoting neuronal survival. Sci. Rep. 6:31158. doi: 10.1038/srep31158, PMID: 27514492 PMC4981891

[ref21] García-Santisteban I. Peters G. J. Giovannetti E. Rodríguez J. A. (2013). USP1 deubiquitinase: cellular functions, regulatory mechanisms and emerging potential as target in cancer therapy. Mol. Cancer 12:91. doi: 10.1186/1476-4598-12-91, PMID: 23937906 PMC3750636

[ref22] Gieffers C. Peters B. H. Kramer E. R. Dotti C. G. Peters J.-M. (1999). Expression of the CDH1-associated form of the anaphase-promoting complex in postmitotic neurons. Proc. Natl. Acad. Sci. USA 96, 11317–11322. doi: 10.1073/pnas.96.20.11317, PMID: 10500174 PMC18031

[ref23] Han T. Jiang S. Zheng H. Yin Q. Xie M. Little M. R.. (2019). Interplay between c-Src and the APC/C co-activator Cdh1 regulates mammary tumorigenesis. Nat. Commun. 10:3716. doi: 10.1038/s41467-019-11618-7, PMID: 31420536 PMC6697746

[ref24] Hendriks I. A. Lyon D. Su D. Skotte N. H. Daniel J. A. Jensen L. J.. (2018). Site-specific characterization of endogenous SUMOylation across species and organs. Nat. Commun. 9:2456. doi: 10.1038/s41467-018-04957-4, PMID: 29942033 PMC6018634

[ref25] Hu Y. Li X. Zhang J. Liu D. Lu R. Li J. D. (2024). A genome-wide CRISPR screen identifies USP1 as a novel regulator of the mammalian circadian clock. FEBS J. 291, 445–457. doi: 10.1111/febs.16990 37909373

[ref26] Ikeuchi Y. Stegmüller J. Netherton S. Huynh M. A. Masu M. Frank D.. (2009). A SnoN–Ccd1 pathway promotes axonal morphogenesis in the mammalian brain. J. Neurosci. 29, 4312–4321. doi: 10.1523/jneurosci.0126-09.2009, PMID: 19339625 PMC2853192

[ref27] IMPC. (n.d.) Gene ANAPC4. International Mouse Phenotyping Consortium. Available at: https://www.mousephenotype.org/data/genes/MGI%3A1098673.

[ref28] Kannan M. Lee S.-J. Schwedhelm-Domeyer N. Nakazawa T. Stegmüller J. (2012). p250GAP is a novel player in the Cdh1-APC/Smurf1 pathway of axon growth regulation. PLoS One 7:e50735. doi: 10.1371/journal.pone.0050735, PMID: 23226367 PMC3511349

[ref29] Kempf M. Clement A. Faissner A. Lee G. Brandt R. (1996). Tau binds to the distal axon early in development of polarity in a microtubule-and microfilament-dependent manner. J. Neurosci. 16, 5583–5592. doi: 10.1523/jneurosci.16-18-05583.1996, PMID: 8795614 PMC6578978

[ref30] Kim A. H. Puram S. V. Bilimoria P. M. Ikeuchi Y. Keough S. Wong M.. (2009). A centrosomal Cdc20-APC pathway controls dendrite morphogenesis in postmitotic neurons. Cell 136, 322–336. doi: 10.1016/j.cell.2008.11.050, PMID: 19167333 PMC2707082

[ref31] Konishi Y. Stegmüller J. Matsuda T. Bonni S. Bonni A. (2004). Cdh1-APC controls axonal growth and patterning in the mammalian brain. Science 303, 1026–1030. doi: 10.1126/science.1093712, PMID: 14716021

[ref32] Kroeger C. M. Ejima K. Hannon B. A. Halliday T. M. McComb B. Teran-Garcia M.. (2021). Persistent confusion in nutrition and obesity research about the validity of classic nonparametric tests in the presence of heteroscedasticity: evidence of the problem and valid alternatives. Am. J. Clin. Nutr. 113, 517–524. doi: 10.1093/ajcn/nqaa357, PMID: 33515017 PMC7948897

[ref33] Kuczera T. Stilling R. M. Hsia H.-E. Bahari-Javan S. Irniger S. Nasmyth K.. (2011). The anaphase promoting complex is required for memory function in mice. Learn. Mem. 18, 49–57. doi: 10.1101/lm.1998411, PMID: 21191042

[ref34] Laemmli U. K. (1970). Cleavage of structural proteins during the assembly of the head of bacteriophage T4. Nature 227, 680–685. doi: 10.1038/227680a0, PMID: 5432063

[ref35] Lasorella A. Stegmüller J. Guardavaccaro D. Liu G. Carro M. S. Rothschild G.. (2006). Degradation of Id2 by the anaphase-promoting complex couples cell cycle exit and axonal growth. Nature 442, 471–474. doi: 10.1038/nature04895, PMID: 16810178

[ref36] Ledvin L. Gassaway B. M. Tawil J. Urso O. Pizzo D. Welsh K. A.. (2023). The anaphase-promoting complex controls a ubiquitination-phosphoprotein axis in chromatin during neurodevelopment. Dev. Cell 58, 2666–2683.e9. doi: 10.1016/j.devcel.2023.10.002, PMID: 37875116 PMC10872926

[ref37] Lee S. B. Kim J. J. Nam H.-J. Gao B. Yin P. Qin B.. (2015). Parkin regulates mitosis and genomic stability through Cdc20/Cdh1. Mol. Cell 60, 21–34. doi: 10.1016/j.molcel.2015.08.011, PMID: 26387737 PMC4592523

[ref38] Lee C. C. Li B. Yu H. Matunis M. J. (2018). Sumoylation promotes optimal APC/C activation and timely anaphase. eLife 7:e29539. doi: 10.7554/elife.29539, PMID: 29517484 PMC5884673

[ref39] Li Z. Zhang B. Yao W. Zhang C. Wan L. Zhang Y. (2019). APC-Cdh1 regulates neuronal apoptosis through modulating glycolysis and pentose-phosphate pathway after oxygen-glucose deprivation and reperfusion. Cell. Mol. Neurobiol. 39, 123–135. doi: 10.1007/s10571-018-0638-x, PMID: 30460429 PMC11469847

[ref40] Liu J. Wan L. Liu J. Yuan Z. Zhang J. Guo J.. (2016). Cdh1 inhibits WWP2-mediated ubiquitination of PTEN to suppress tumorigenesis in an APC-independent manner. Cell Discov. 2:15044. doi: 10.1038/celldisc.2015.44, PMID: 27462441 PMC4860961

[ref41] López-Murcia F. J. Reim K. Jahn O. Taschenberger H. Brose N. (2019). Acute Complexin knockout abates spontaneous and evoked transmitter release. Cell Rep. 26, 2521–2530.e5. doi: 10.1016/j.celrep.2019.02.030, PMID: 30840877

[ref42] Maestre C. Delgado-Esteban M. Gomez-Sanchez J. C. Bolaños J. P. Almeida A. (2008). Cdk5 phosphorylates Cdh1 and modulates cyclin B1 stability in excitotoxicity. EMBO J. 27, 2736–2745. doi: 10.1038/emboj.2008.195, PMID: 18818692 PMC2572178

[ref43] Malureanu L. Jeganathan K. B. Jin F. Baker D. J. van Ree J. H. Gullon O.. (2010). Cdc20 hypomorphic mice fail to counteract de novo synthesis of cyclin B1 in mitosis. J. Cell Biol. 191, 313–329. doi: 10.1083/jcb.201003090, PMID: 20956380 PMC2958469

[ref44] Man C. Rosa J. Yip Y. L. Cheung A. L.-M. Kwong Y. L. Doxsey S. J.. (2008). Id1 overexpression induces tetraploidization and multiple abnormal mitotic phenotypes by modulating Aurora a. Mol. Biol. Cell 19, 2389–2401. doi: 10.1091/mbc.e07-09-0875, PMID: 18353975 PMC2397319

[ref45] Mata G. Cuesto G. Heras J. Morales M. Romero A. Rubio J. (2017). Biomedical engineering systems and technologies, 9th international joint conference, BIOSTEC 2016, Rome, Italy, February 21–23, 2016, revised selected papers. Commun. Comput. Inf. Sci., 41–55.

[ref46] Mathieson T. Franken H. Kosinski J. Kurzawa N. Zinn N. Sweetman G.. (2018). Systematic analysis of protein turnover in primary cells. Nat. Commun. 9:689. doi: 10.1038/s41467-018-03106-1, PMID: 29449567 PMC5814408

[ref47] Matic I. Schimmel J. Hendriks I. A. van de Rijke F. van Dam H. Gnad F.. (2010). Site-specific identification of SUMO-2 targets in cells reveals an inverted SUMOylation motif and a hydrophobic cluster SUMOylation motif. Mol. Cell 39, 641–652. doi: 10.1016/j.molcel.2010.07.026, PMID: 20797634

[ref48] Meijering E. Jacob M. Sarria J. -C. F. Steiner P. Hirling H. Unser M. (2004). Design and validation of a tool for neurite tracing and analysis in fluorescence microscopy images. Cytometry A 58A, 167–176. doi: 10.1002/cyto.a.20022, PMID: 15057970

[ref49] Oh E. Akopian D. Rape M. (2018). Principles of ubiquitin-dependent signaling. Annu. Rev. Cell Dev. Biol. 34, 137–162. doi: 10.1146/annurev-cellbio-100617-062802 30110556

[ref50] Peters J.-M. (2006). The anaphase promoting complex/cyclosome: a machine designed to destroy. Nat. Rev. Mol. Cell Biol. 7, 644–656. doi: 10.1038/nrm1988, PMID: 16896351

[ref51] Preibisch S. Saalfeld S. Tomancak P. (2009). Globally optimal stitching of tiled 3D microscopic image acquisitions. Bioinformatics 25, 1463–1465. doi: 10.1093/bioinformatics/btp184, PMID: 19346324 PMC2682522

[ref52] Reim K. Mansour M. Varoqueaux F. McMahon H. T. Südhof T. C. Brose N.. (2001). Complexins regulate a late step in Ca2+−dependent neurotransmitter release. Cell 104, 71–81. doi: 10.1016/s0092-8674(01)00192-1 11163241

[ref53] Reim K. Wegmeyer H. Brandstätter J. H. Xue M. Rosenmund C. Dresbach T.. (2005). Structurally and functionally unique complexins at retinal ribbon synapses. J. Cell Biol. 169, 669–680. doi: 10.1083/jcb.200502115, PMID: 15911881 PMC2171701

[ref54] Schimmel J. Eifler K. Sigurðsson J. O. Cuijpers S. A. G. Hendriks I. A. Verlaan-de Vries M.. (2014). Uncovering SUMOylation dynamics during cell-cycle progression reveals FoxM1 as a key mitotic SUMO target protein. Mol. Cell 53, 1053–1066. doi: 10.1016/j.molcel.2014.02.001 24582501

[ref55] Schindelin J. Arganda-Carreras I. Frise E. Kaynig V. Longair M. Pietzsch T.. (2012). Fiji: an open-source platform for biological-image analysis. Nat. Methods 9, 676–682. doi: 10.1038/nmeth.2019 22743772 PMC3855844

[ref56] Sholl D. A. (1953). Dendritic organization in the neurons of the visual and motor cortices of the cat. J. Anat. 87, 387–406. PMID: 13117757 PMC1244622

[ref57] Simoneau A. Engel J. L. Bandi M. Lazarides K. Liu S. Meier S. R.. (2023). Ubiquitinated PCNA drives USP1 synthetic lethality in cancer. Mol. Cancer Ther. 22, 215–226. doi: 10.1158/1535-7163.MCT-22-0409, PMID: 36228090 PMC9891357

[ref58] Skovlund E. Fenstad G. U. (2001). Should we always choose a nonparametric test when comparing two apparently nonnormal distributions? J. Clin. Epidemiol. 54, 86–92. doi: 10.1016/s0895-4356(00)00264-x, PMID: 11165471

[ref59] Stegmüller J. Huynh M. A. Yuan Z. Konishi Y. Bonni A. (2008). TGFβ-Smad2 signaling regulates the Cdh1-APC/SnoN pathway of axonal morphogenesis. J. Neurosci. 28, 1961–1969. doi: 10.1523/jneurosci.3061-07.2008, PMID: 18287512 PMC6671436

[ref60] Stegmüller J. Konishi Y. Huynh M. A. Yuan Z. DiBacco S. Bonni A. (2006). Cell-intrinsic regulation of axonal morphogenesis by the Cdh1-APC target SnoN. Neuron 50, 389–400. doi: 10.1016/j.neuron.2006.03.034, PMID: 16675394

[ref61] Tavares G. Martins M. Correia J. S. Sardinha V. M. Guerra-Gomes S. das Neves S. P.. (2017). Employing an open-source tool to assess astrocyte tridimensional structure. Brain Struct. Funct. 222, 1989–1999. doi: 10.1007/s00429-016-1316-8, PMID: 27696155 PMC5406431

[ref9001] Tirard M. Hsiao H.-H. Nikolov M. Urlaub H. Melchior F. Brose N.. (2012). In vivo localization and identification of SUMOylated proteins in the brain of His6-HA-SUMO1 knock-in mice. Proc. Natl. Acad. Sci. 109, 21122–21127. doi: 10.1073/pnas.1215366110, PMID: 23213215 PMC3529052

[ref62] Thornton B. R. Ng T. M. Matyskiela M. E. Carroll C. W. Morgan D. O. Toczyski D. P. (2006). An architectural map of the anaphase-promoting complex. Genes Dev. 20, 449–460. doi: 10.1101/gad.1396906, PMID: 16481473 PMC1369047

[ref63] Tomomori-Sato C. Sato S. Conaway R. C. Conaway J. W. (2013). Gene regulation, methods and protocols. Methods Mol. Biol. 977, 273–287. doi: 10.1007/978-1-62703-284-1_22, PMID: 23436370 PMC3693849

[ref64] Towbin H. Staehelin T. Gordon J. (1979). Electrophoretic transfer of proteins from polyacrylamide gels to nitrocellulose sheets: procedure and some applications. *Proc. Natl. Acad. Sci.* USA 76, 4350–4354. doi: 10.1073/pnas.76.9.4350, PMID: 388439 PMC411572

[ref65] Tran K. Kamil J. P. Coen D. M. Spector D. H. (2010). Inactivation and disassembly of the anaphase-promoting complex during human cytomegalovirus infection is associated with degradation of the APC5 and APC4 subunits and does not require UL97-mediated phosphorylation of Cdh1. J. Virol. 84, 10832–10843. doi: 10.1128/jvi.01260-10, PMID: 20686030 PMC2950577

[ref66] Wan L. Chen M. Cao J. Dai X. Yin Q. Zhang J.. (2017). The APC/C E3 ligase complex activator FZR1 restricts BRAF oncogenic function. Cancer Discov. 7, 424–441. doi: 10.1158/2159-8290.cd-16-0647, PMID: 28174173 PMC5380472

[ref67] Wan L. Zou W. Gao D. Inuzuka H. Fukushima H. Berg A. H.. (2011). Cdh1 regulates osteoblast function through an APC/C-independent modulation of Smurf1. Mol. Cell 44, 721–733. doi: 10.1016/j.molcel.2011.09.024, PMID: 22152476 PMC3240853

[ref68] Watanabe Y. Khodosevich K. Monyer H. (2014). Dendrite development regulated by the schizophrenia-associated gene FEZ1 involves the ubiquitin proteasome system. Cell Rep. 7, 552–564. doi: 10.1016/j.celrep.2014.03.022 24726361

[ref69] Wiebusch L. Bach M. Uecker R. Hagemeier C. (2005). Human cytomegalovirus inactivates the G0/G1-APC/C ubiquitin ligase by Cdh1 dissociation. Cell Cycle 4, 1435–1439. doi: 10.4161/cc.4.10.2077, PMID: 16138013

[ref70] Wirth K. G. Ricci R. Giménez-Abián J. F. Taghybeeglu S. Kudo N. R. Jochum W.. (2004). Loss of the anaphase-promoting complex in quiescent cells causes unscheduled hepatocyte proliferation. Genes Dev. 18, 88–98. doi: 10.1101/gad.285404, PMID: 14724179 PMC314282

[ref71] Xue M. Stradomska A. Chen H. Brose N. Zhang W. Rosenmund C.. (2008). Complexins facilitate neurotransmitter release at excitatory and inhibitory synapses in mammalian central nervous system. Proc. Natl. Acad. Sci. USA 105, 7875–7880. doi: 10.1073/pnas.0803012105, PMID: 18505837 PMC2409395

[ref72] Yang Y. Kim A. H. Yamada T. Wu B. Bilimoria P. M. Ikeuchi Y.. (2009). A Cdc20-APC ubiquitin signaling pathway regulates presynaptic differentiation. Science 326, 575–578. doi: 10.1126/science.1177087, PMID: 19900895 PMC2846784

[ref73] Yatskevich S. Kroonen J. S. Alfieri C. Tischer T. Howes A. C. Clijsters L.. (2021). Molecular mechanisms of APC/C release from spindle assembly checkpoint inhibition by APC/C SUMOylation. Cell Rep. 34:108929. doi: 10.1016/j.celrep.2021.108929, PMID: 33789095 PMC8028313

