# Supplementary material for: Deletion of a core APC/C component reveals APC/C function in regulating neuronal USP1 levels and morphology
Source: Front Mol Neurosci. 2024 Jun 12;17:1352782. doi: 10.3389/fnmol.2024.1352782 (PMC11199872; doi:10.3389/fnmol.2024.1352782)
Supplement: Supplementary file 1 [file Data_Sheet_1.PDF]

Supplementary Material

Deletion of a core APC/C component reveals APC/C function in regulating neuronal USP1 levels and cell morphology

Jennifer L. Day, Marilyn Tirard, and Nils Brose*

*** Correspondence:** Corresponding Author: brose@mpinat.mpg.de

1 Supplementary Figures and Tables

1.1 Supplementary Figures

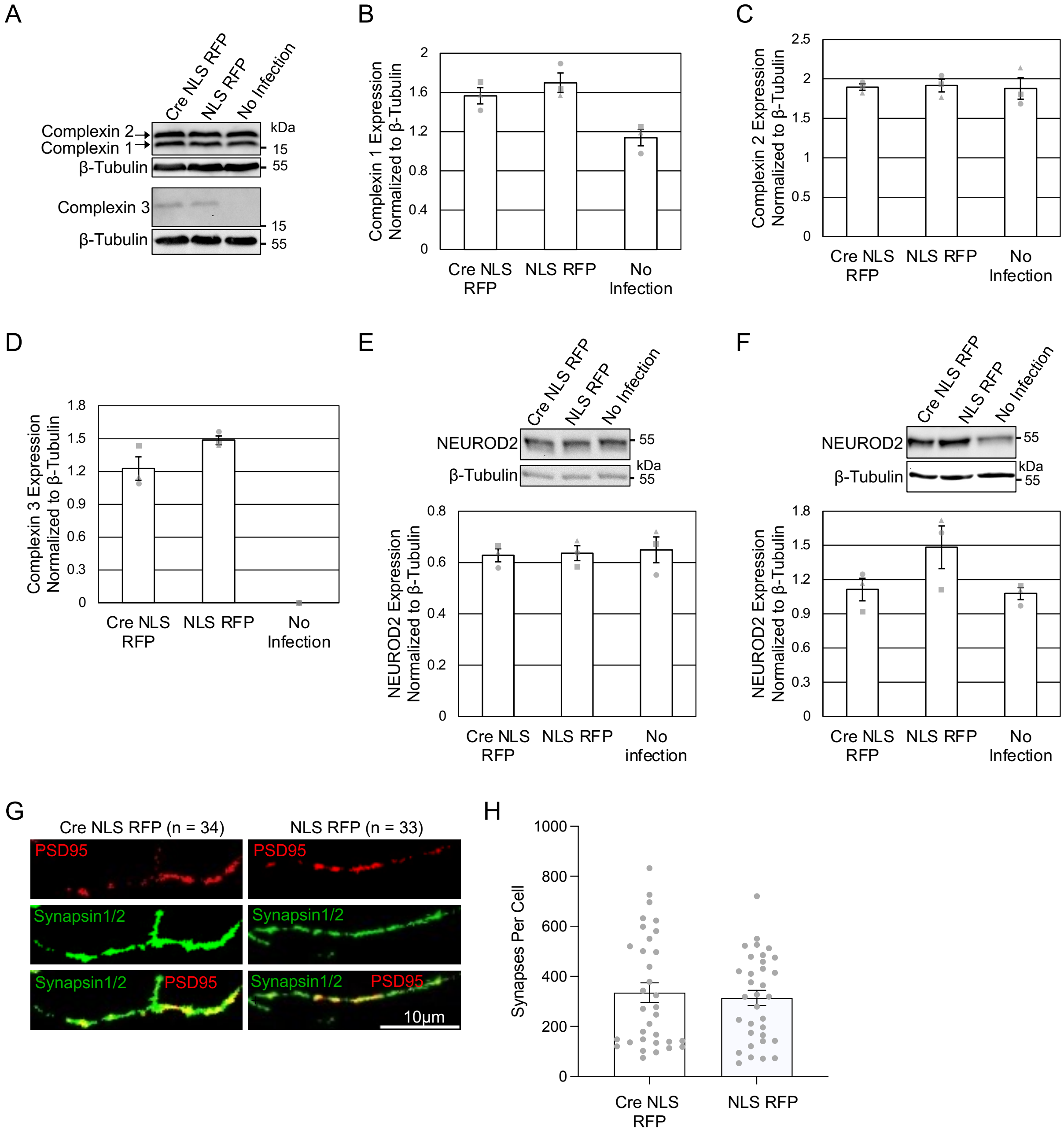

Supplementary Figure 1. APC4 loss does not affect synaptogenesis. **(A-F)** Cortical cultures were prepared from *ANAPC4* cKO mice and infected at DIV1 with Cre NLS RFP- or NLS RFP-expressing lentivirus. Neurons were harvested at DIV5 or DIV11. These lysates were also used in Figures 3 and 4, so APC4 loss is shown in Figures 3A and 4A. Protein quantification was done by averaging values of three independent experiments (representative experiments displayed). Experiments 1, 2, and 3 are represented by a circle, triangle, and square respectively. Error bars: SEM. **(A)** WB of DIV11 lysates shows Complexin 1-3 and β-Tubulin. Arrows: Complexin paralogs. **(B)** Bar graph depicts the average Complexin 1 levels normalized to β-Tubulin. There was no significant difference in Complexin 1 levels between infected samples ($t(2) = -1.021$, $p = 0.365$). **(C)** The bar graph depicts the average Complexin 2 levels normalized to β-Tubulin. There was no significant difference in Complexin 2 levels between infected samples ($t(2) = -0.217$, $p = 0.839$). **(D)** The bar graph depicts the average levels of Complexin 3 normalized to β-Tubulin. There was no significant difference in Complexin 3 levels between CRE NLS RFP- AND NLS RFP-infected samples ($t(2) = -1.704$, $p = 0.163$). **(E)** WB of DIV5 lysates show NEUROD2 and β-Tubulin. The bar graph depicts the average quantity of NEUROD2 normalized to β-Tubulin. Only the upper and more intense NEUROD2 band was quantified. There was no significant difference in NEUROD2 levels between infected samples ($t(2) = -0.215$, $p = 0.841$). **(F)** WB of DIV11 lysates shows NEUROD2 and β-Tubulin. The bar graph depicts NEUROD2 normalized to β-Tubulin. There was no significant difference between Cre- and control-infected samples ($t(2) = -1.753$, $p = 0.155$). **(G-H)** Primary cortical neuron cultures prepared from *ANAPC4* cKO mice were infected at DIV1 with Cre NLS RFP- ($n = 34$) or NLS RFP-expressing ($n = 33$) lentivirus, and the neurons were fixed at DIV11 and immunolabeled with PSD95 (red) and Synapsin1/2 (green) antibodies. **(G)** Representative images of neurites from neurons infected with Cre NLS RFP or NLS RFP lentivirus indicate the localization of PSD95 and Synapsin1/2, and merged images show co-localized puncta. Scale bar: 10 μm. **(H)** The bar graph depicts the average total number of synapses, corresponding to puncta double-positive for post-synaptic (PSD95) and pre-synaptic (Synapsin1/2) markers. There was no difference in the number of synapses between infected samples as assessed by Welch's t-test ($t(62.382) = -0.479$, $p = 0.634$). Circles: individual data points.

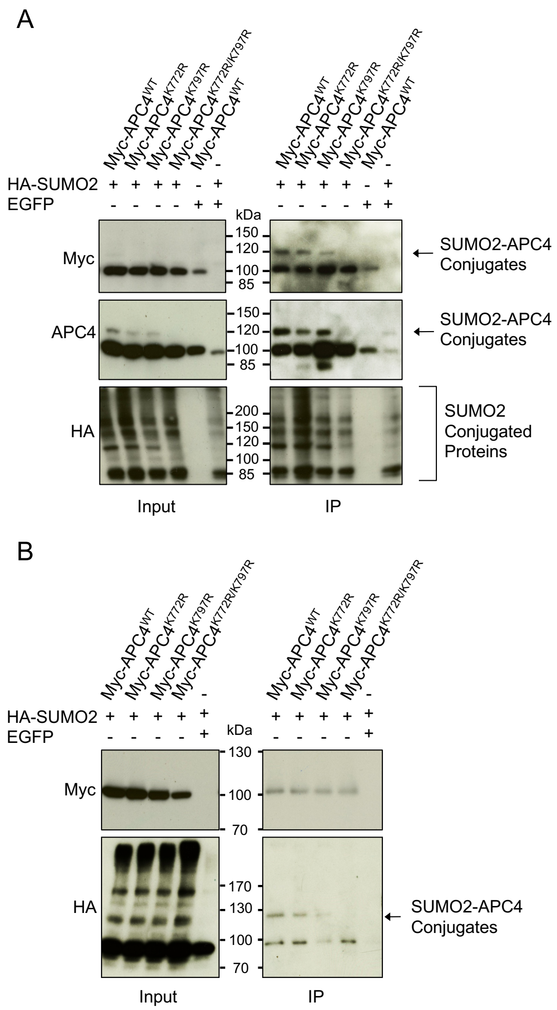

Supplementary Figure 2. Mouse APC4 is SUMOylated on lysines 772 and 797. **(A-B)** WB analysis of Input (left) and HA-IP eluates (right) from HEK293 cells transfected with the indicated APC4 and SUMO2 overexpression plasmids, including APC4 constructs with single or double mutations of lysines 772 and 798. **(A)** WB shows Myc-tagged proteins, APC4, and HA-tagged SUMO2 upon HA-IP in the presence of NEM (representative experiment). Arrows, APC4-SUMO2 conjugates; bracket, all SUMO2 conjugation. **(B)** WB shows Myc-tagged proteins and HA-SUMO2 upon Myc-IP in the absence of NEM. The arrow indicates SUMOylated APC4.

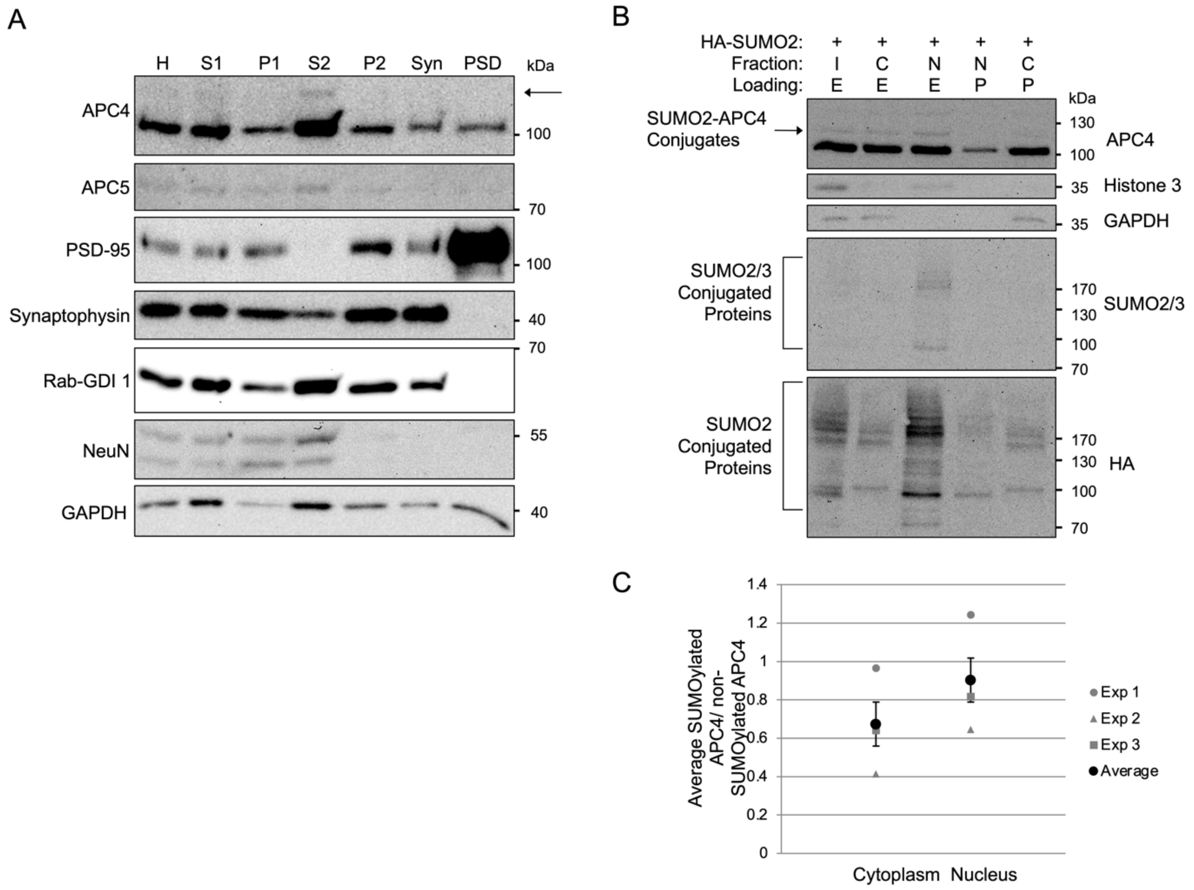

Supplementary Figure 3. SUMOylated APC4 is enriched in the cytosol fraction of the mouse cortex and in the nuclear fraction of HEK293FT cells. **(A)** WB analysis of the indicated proteins in subcellular fractions of adult mouse cortex prepared in the absence of NEM. H, homogenate; S1, supernatant after P1 sedimentation; P1, nuclear pellet; S2, supernatant after P2 sedimentation; P2, crude synaptosome pellet; Syn, synaptosomes; PSD, crude PSD. APC4 was detected in all fractions tested. The arrow indicates SUMOylated APC4, which was enriched in the cytosolic (S2) fraction. **(B)** HEK293 cells were transfected with HA-SUMO2, lysed in the presence of NEM (I, input), and fractionated into nuclear (N) and cytosolic (C) fractions. To quantify protein levels, three separate experiments were conducted. Representative blots are shown for the indicated antibodies. Each lane contains either equal amounts of protein loading (E) or an equal percentage of the cell volume (P). The arrow indicates conjugates of SUMO2-APC4 and the brackets depict total SUMO conjugation. **(C)** Dot plot depicting the quantification of the amount of APC4 conjugated to one SUMO moiety in each fraction after normalization to the total amount of non-SUMOylated APC4. The experimental averages (dark circle) were calculated using the ratios of protein in the E lanes, and the average difference for all three independent experiments was calculated. Error bars, SEM. Experiments 1, 2, and 3 are represented by a light circle, triangle, and square respectively. The average difference value for all experiments was compared to a predicted value of 0 in a paired t-test ($t(2)$:7.755 , $p = 0.008$).

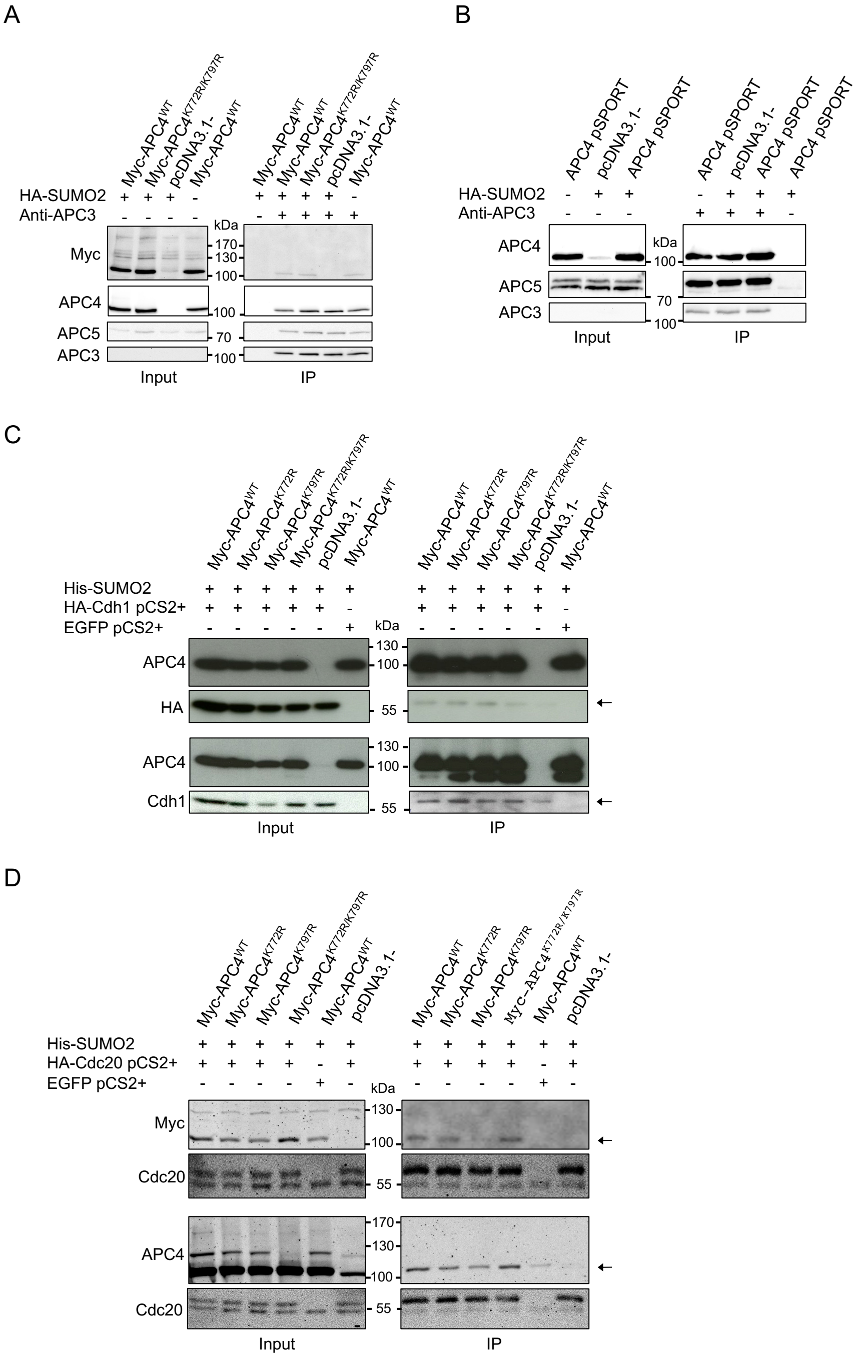

Supplementary Figure 4. APC4 SUMOylation does not affect the formation of the APC/C or the binding of APC/C activators, Cdh1 or Cdc20. **(A-D)** Representative WB of Input (left) and APC3-IP eluates (right) obtained from HEK293 cells transfected with HA-SUMO2 and APC4 overexpression constructs and lysed without NEM. **(A)** WB indicates Myc, APC4, APC5, and APC3 expression upon APC3-IP of input and eluates obtained from HEK293 cells transfected with HA-SUMO2, Myc-APC4^{WT} (wildtype), and Myc-APC4$^{K772R/K797R}$ (SUMOylation-deficient) overexpression constructs. **(B)** WB analysis indicates APC4, APC5, and APC3 expression upon APC3-IP of input and eluates obtained from HEK293 cells transfected with HA-SUMO2 and a non-tagged wildtype APC4 construct. **(C)** WB analysis indicates APC4, HA, and Cdh1 expression

upon Myc-IP of input and eluates obtained from HEK293 cells transfected with HA-Cdh1, His-SUMO2, and either Myc-APC4^{WT} (wildtype) or a Myc-APC4 construct that has a single or a double mutation of lysines 772 and 798 (representative experiment). The arrows indicate Cdh1 bound to the APC/C. **(D)** WB analysis indicates Myc, Cdc20, and APC4 expression upon HA-IP of input and eluates obtained from HEK293 cells transfected with HA-Cdc20, His-SUMO2, and either Myc-APC4^{WT} (wildtype) or a Myc-APC4 construct that possess single or a double mutation of lysines 772 and 798 (representative experiment). Arrow indicate Cdc20 bound to the APC/C.

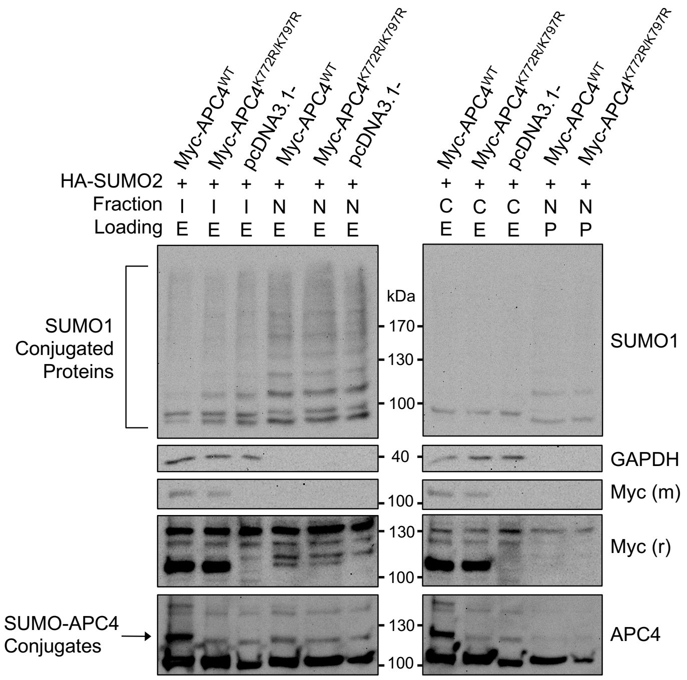

Supplementary Figure 5. APC4 SUMOylation does not affect the subcellular localization of APC4. HEK293 cells were transfected with the indicated constructs, including APC4 constructs with single or double mutations of lysines 772 and 798. Cells were lysed in the presence of NEM (I) and fractionated into nuclear (N) and cytosolic (C) fractions. WB was conducted to detect SUMO1, GAPDH, Myc (mouse, m), Myc (rabbit, r), and APC4. Each lane contains either equal amounts of protein loading (E) or an equal percentage of the cell volume (P). Arrow, SUMO-APC4 conjugates; bracket, total SUMO1 conjugation.

1.2 Supplementary Tables

Supplementary Table 1. Transgenic mice used

**Mouse line**	**Genotype**	**Provider**
ANAPC4 tm1a	KO-first	EUCOMM #1098673
ANAPC4 tm1c	ANAPC4 cKO	Crossed *tm1a/tm1a* to FLIR
ANAPC4 tm1d	*ANAPC4* KO	Infected *tm1c/tm1c* neurons with lentivirus expressing Cre
FLIR	Flp recombinase driven by the *Gt(ROSA)26Sor* promoter	JAX #003946

Supplementary Table 2. Mouse genotyping

#	Primer purpose	Primer Sequence	PCR primers used: PCR product size
1	Actin control	5'-TGTGGCTTTCTGAACTTGACA-3'	1 and 2: 119bp
2	Actin control	5'-ACCAGAGGCATACAGGGACA-3'	
3	FLIR	5'-FAM-GTGACAGAGACAAAGACAAGCGTTAG-3'	3 and 4: 139bp
4	FLIR	5'-AATTGCCGGTCCTATTTACTCGTT-3'	
5	*tm1a* and *tm1c* forward	5'-CCCCTCATGAAGAACTACAGG-3'.	5 and 6: 316bp (wildtype)
6	*tm1c* rev	5'-ATCGCTTTTGCCTTGACG-3'	5 and 6: 512bp (*tm1c*)
7	*tm1a* rev	5'- CACCCAACTGACCTTGGGCAAG-3'.	5 and 7: 455bp (*tm1a*)

Supplementary Table 3. DNA and plasmids acquired in this study

Plasmid or DNA	Source	Experimental purpose
Mouse APC4 (Gene ID: 52206) cDNA in pCMV6-Kan/Neo	OriGene #MC206344	Cloning APC4 constructs
pcDNA3.1	Invitrogen	Transfection constructs
f(syn)w-rbn-iCreRFP (CRE NLS RFP)	kind gift of C. Rosenmund (Berlin)	Lentiviral constructs
f(syn)w-rbn-RFP (NLS RFP)	kind gift of C. Rosenmund (Berlin)	Lentiviral constructs
(syn)w-iCreRFP-P2A (CRE RFP P2A)	kind gift of C. Rosenmund (Berlin)	Lentivirus constructs
pCMVdeltaR8.2	Addgene #12263	lentiviral supplementary proteins
pVSV-G	Addgene #12259	lentiviral supplementary proteins
pEGFP-N1	Clontech	Cloning, transfections
HA SUMO2 pCRUZ	kind gift of F. Melchior (Heidelberg)	Transfection constructs
His SUMO2 pCRUZ	kind gift of F. Melchior (Heidelberg)	Transfection constructs
HA Cdh1 pCS2+	kind gift of M. Kirschner (Boston)	Transfection constructs
HA Cdc20 pCS2+	kind gift of M. Kirschner (Boston)	Transfection constructs

Supplementary Table 4. Primary antibodies used in this study

Name	Manufacturer	Catalogue #	Source	Dilution WB	Dilution ICC
APC4	Novus Biologicals	A2095	Rabbit	1:2000	1:500
APC4	Santa Cruz	sc-514895	Mouse		1:200
APC3 (Cdc27)	BD Biosciences	610455	Mouse	1:300; 2.5μg/IP	
APC5	Bethyl	A301-026A-M	Rabbit	1:500	
β Tubulin 2.1	Sigma	T4026	Mouse	1:2000	
β III-Tubulin	Synaptic Systems	302 304	Guinea pig		1:2000
Cdc20	Santa Cruz	sc-13162	Mouse	1:200	
Cdh1	Sigma	C7855	Mouse	1:1000	
Complexin 1/2	Synaptic Systems	122	Rabbit	1:4000	
Complexin 3	Synaptic Systems	122 302	Rabbit	1:2000	
Cre	Synaptic Systems	257 003	Rabbit		1:2000
Cyclin B1 (GNS1)	Santa Cruz	sc-245	Mouse	1:200	
FEZ1	J. Chua gift	Homemade	Rabbit	1:1000	
GAPDH	Abcam	ab8245	Mouse	1:2000	
HA	Biolegend	MMS-101R-500	Mouse	1:2000	
Histone 3	Abcam; K. Nave gift	ab18521	Rabbit	1:1000	
MAP2	Novus Biologicals	NB300-213	Chicken		1:600
Myc (used unless specified)	Sigma	C3956	Rabbit	1:2000	
Myc	Sigma	M5546	Mouse	1:2000	
NeuN	Millipore	MAB377	Mouse	1:1000	
NeuroD2	Abcam	ab109406	Rabbit	1:500	
PSD95	Abcam	ab2723	Mouse	1:2000	1:200
Rab GDI	Synaptic Systems	130 011	Mouse	1:2000	
RFP	Synaptic Systems	390 004	Guinea pig		1:2000
SMI-312	HISS Diagnostics	SMI-312R	Mouse		1:1000
SnoN (SKIL)	Proteintech	19218-1AP	Rabbit	1:1000	
SUMO1 (M. Tirard generated)	Iowa Hybridoma Bank, Iowa	21C7	Mouse	1:100	
SUMO2/3	Abcam	ab81371	Mouse	1:1000	
Synapsin 1/2	Synaptic Systems	106 002	Rabbit	1:2000	1:1000
Synaptophysin (p611)	Made by N. Brose	Homemade	Rabbit	1:2000	
USP1	Cell Signaling	8033S	Rabbit	1:1000	

Supplementary Table 5. Secondary antibodies used in this study

Name	Manufacturer	Catalogue #	Dilution WB	Dilution ICC
Anti-mouse HRP	Biorad	#172-1011	1:5000	
Anti-rabbit HRP	Biorad	#172-1019	1:5000	
Peroxidase AffiniPure Goat Anti-Mouse IgG H+L HRP	Jackson Immuno Research	#115-035-146	1:5000	
Peroxidase AffiniPure Goat Anti-Rabbit IgG H+L HRP	Jackson Immuno Research	#111-035-144	1:5000	
Anti-chicken IgG Alexa 405	Abcam	#ab175674		1:1000
Anti-chicken IgG Alexa 633	Thermo Fisher	#A-21103		1:1000
Anti-guinea pig IgG Alexa 555	Thermo Fisher	#A-21435		1:1000
Anti-guinea pig IgG Alexa 633	MoBiTec	#A21105		1:1000
Anti-mouse IgG Alexa 488	Thermo Fisher	#A-11029		1:1000
Anti-mouse IgG Alexa 633	Thermo Fisher,	#A-21052		1:1000
Anti-rabbit IgG Alexa 488	Thermo Fisher	#A-11008		1:1000
